# Supplementary figures and images for: HSV-1 and influenza infection induce linear and circular splicing of the long NEAT1 isoform
Source: PLoS One. 2022 Oct 24;17(10):e0276467. doi: 10.1371/journal.pone.0276467 (PMC9591066; doi:10.1371/journal.pone.0276467)

**a**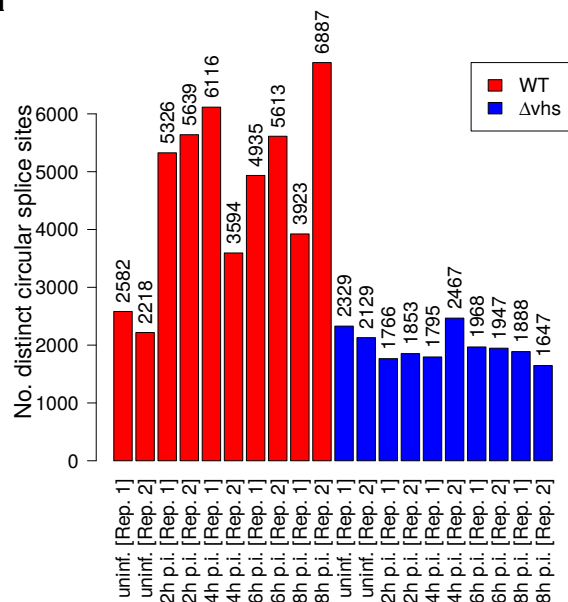**b**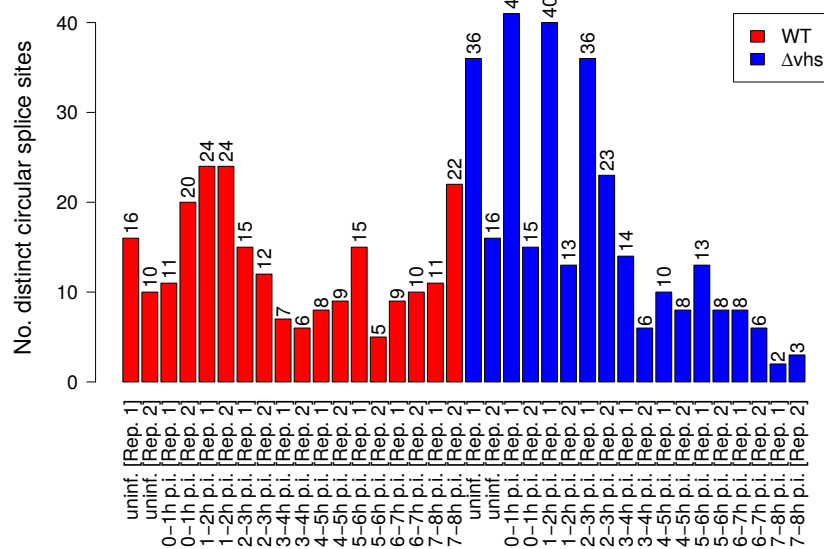**c**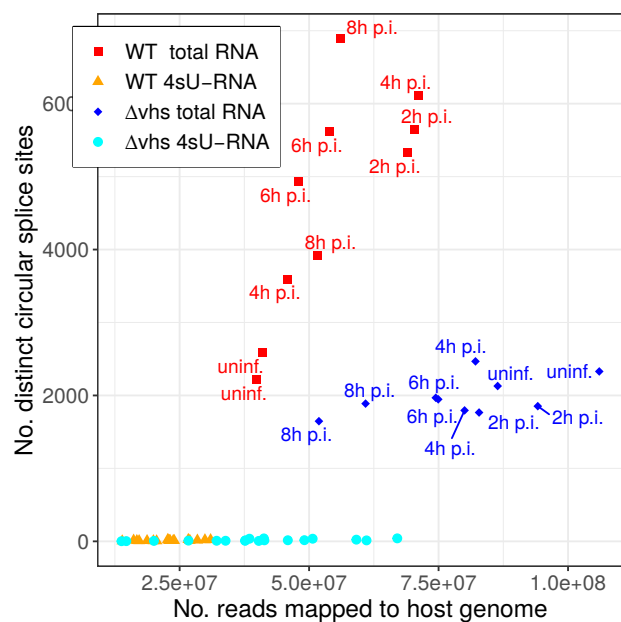**d**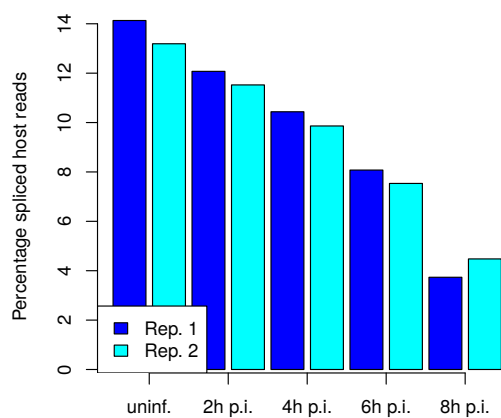**e**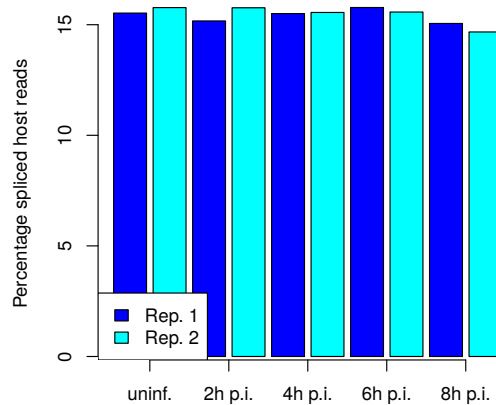**S1 Fig**

Supplement: S1 Fig — (a, b) Number of circRNAs identified for each sample of (a) total RNA and (b) 4sU-RNA with ≥ 2 reads per sample. (c) Scatterplot comparing the number of reads mapped to the human genome against the number of distinct circular splice sites identified by the de novo circRNA detection approach outlined in Fig 2A for the total RNA and 4sU-RNA time-courses in HSV-1 WT and Δvhs infection. Numbers are shown separately for each replicate. (d, e) Percentage spliced host reads (= no. reads aligning to known splice junctions of host protein-coding genes / no. of mapped host reads × 100) for both replicates of total RNA-seq for (d) WT and (e) Δvhs infection. (PDF) [file pone.0276467.s001.pdf]

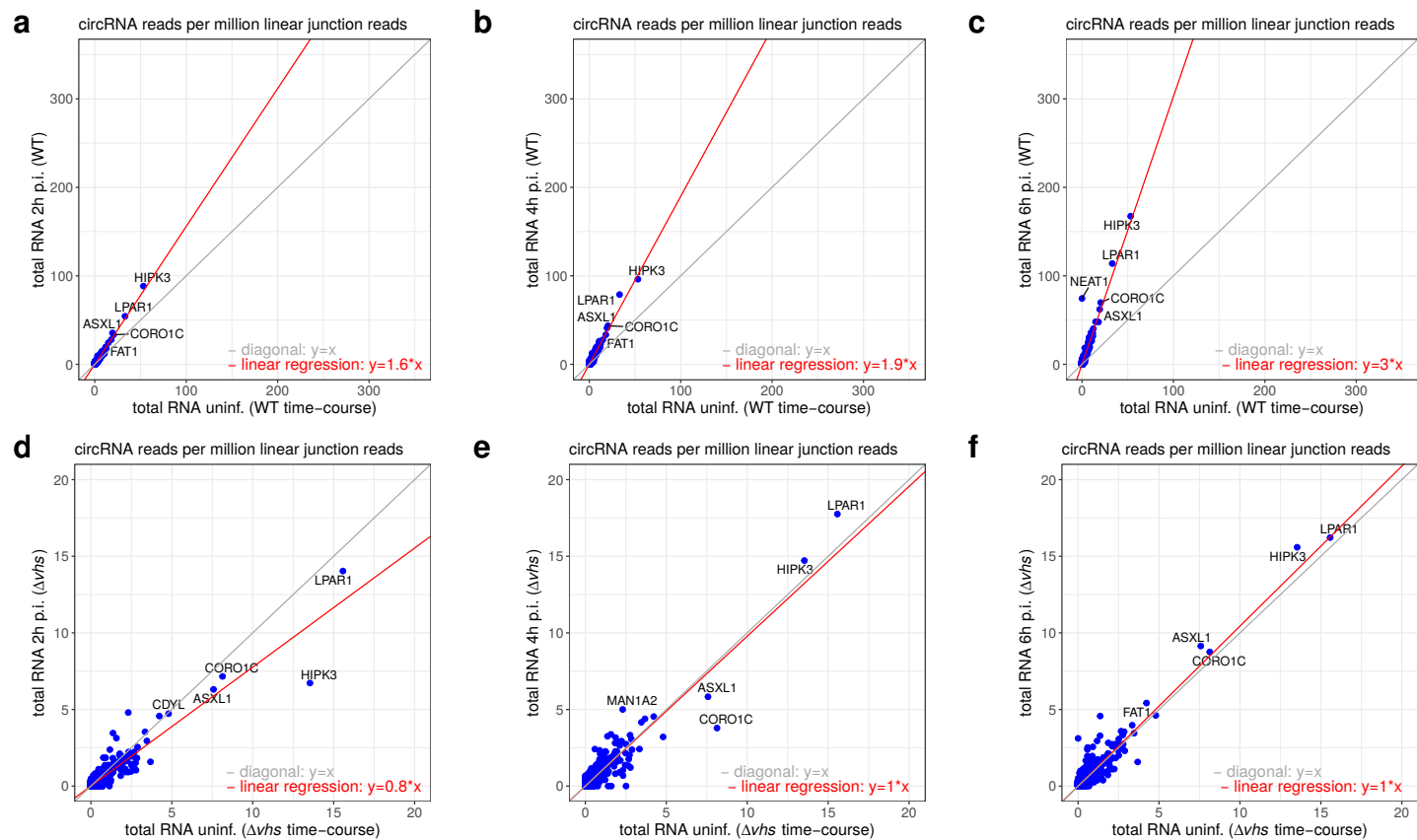

**S2 Fig**

Supplement: S2 Fig — Scatterplots comparing normalized circRNA counts (normalized to the number of linear junction reads mapped to the host genome) between mock and 2, 4 and 6 h p.i. WT infection (a-c) and mock and 2, 4 and 6 h p.i. Δvhs infection (d-f). Linear regression analysis across all circRNAs (red line) was used to estimate the enrichment of circRNAs relative to linear mRNAs in HSV-1 infection compared to mock infection. The regression estimate for the enrichment is shown on the bottom right. The gray line indicates the diagonal, i.e., equal values on the x- and y-axis. The five most highly expressed circRNAs are marked by name. Corresponding scatterplots for 8 h p.i. are shown in Fig 2C and 2D. (PDF) [file pone.0276467.s002.pdf]

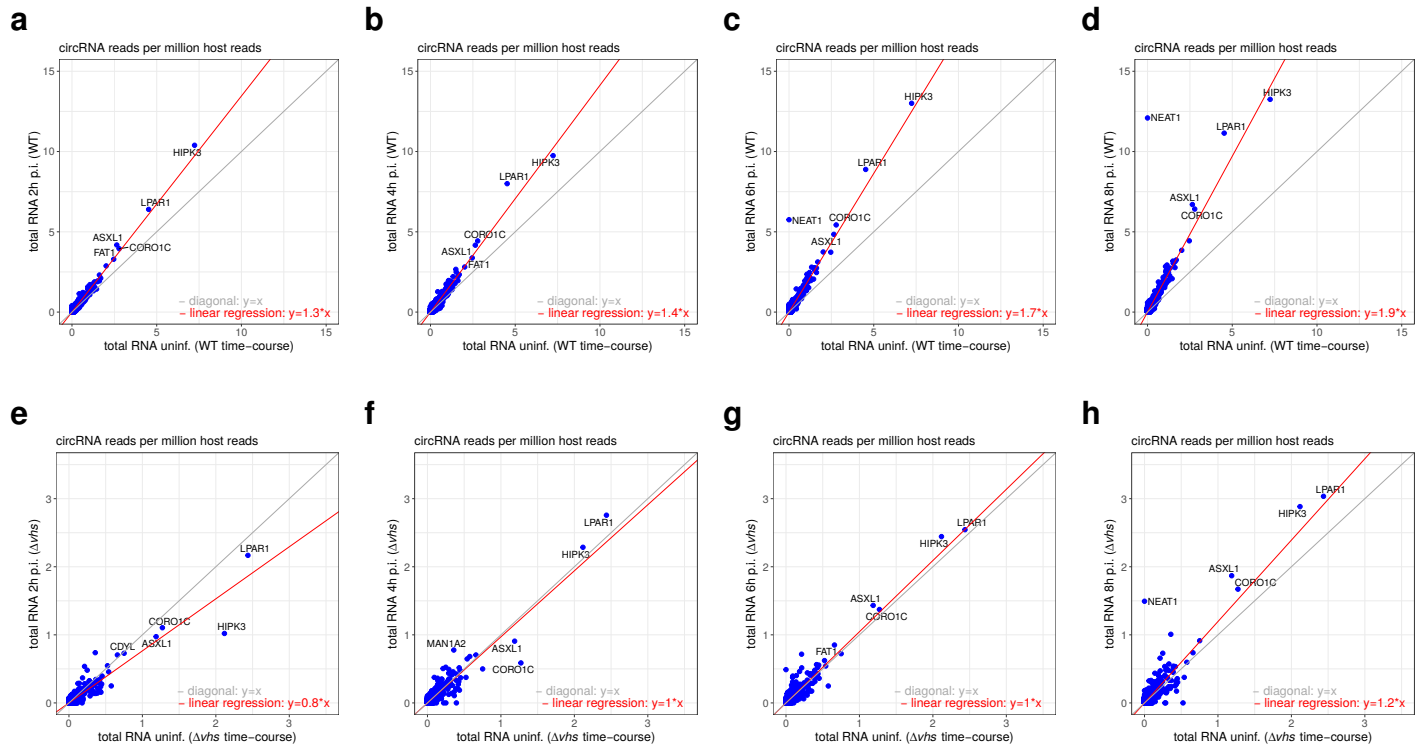

**S3 Fig**

Supplement: S3 Fig — Scatterplots comparing normalized circRNA counts normalized to the total number of reads mapped to the host genome between mock and 2, 4, 6 and 8 h p.i. WT infection (a-d) and mock and 2, 4, 6 and 8 h p.i. Δvhs infection (e-h). Linear regression analysis across all circRNAs (red line) was used to estimate the enrichment of circRNAs relative to all host reads in HSV-1 infection compared to mock infection. The regression estimate for the enrichment is shown on the bottom right. The gray line indicates the diagonal, i.e., equal values on the x- and y-axis. The five most highly expressed circRNAs are marked by name. (PDF) [file pone.0276467.s003.pdf]

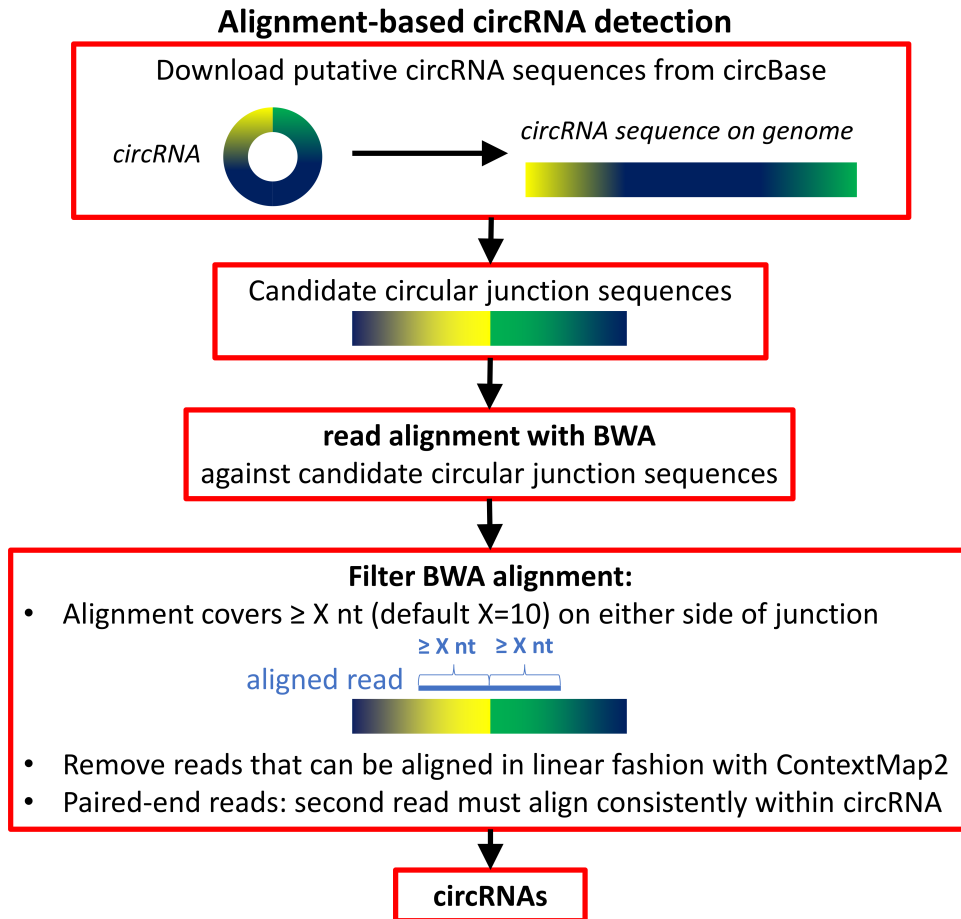

**S4 Fig**

Supplement: S4 Fig — For details see Materials and Methods. This approach allows increasing or decreasing the required overlap of a read with either side of a circular junction (=: X). (PDF) [file pone.0276467.s004.pdf]

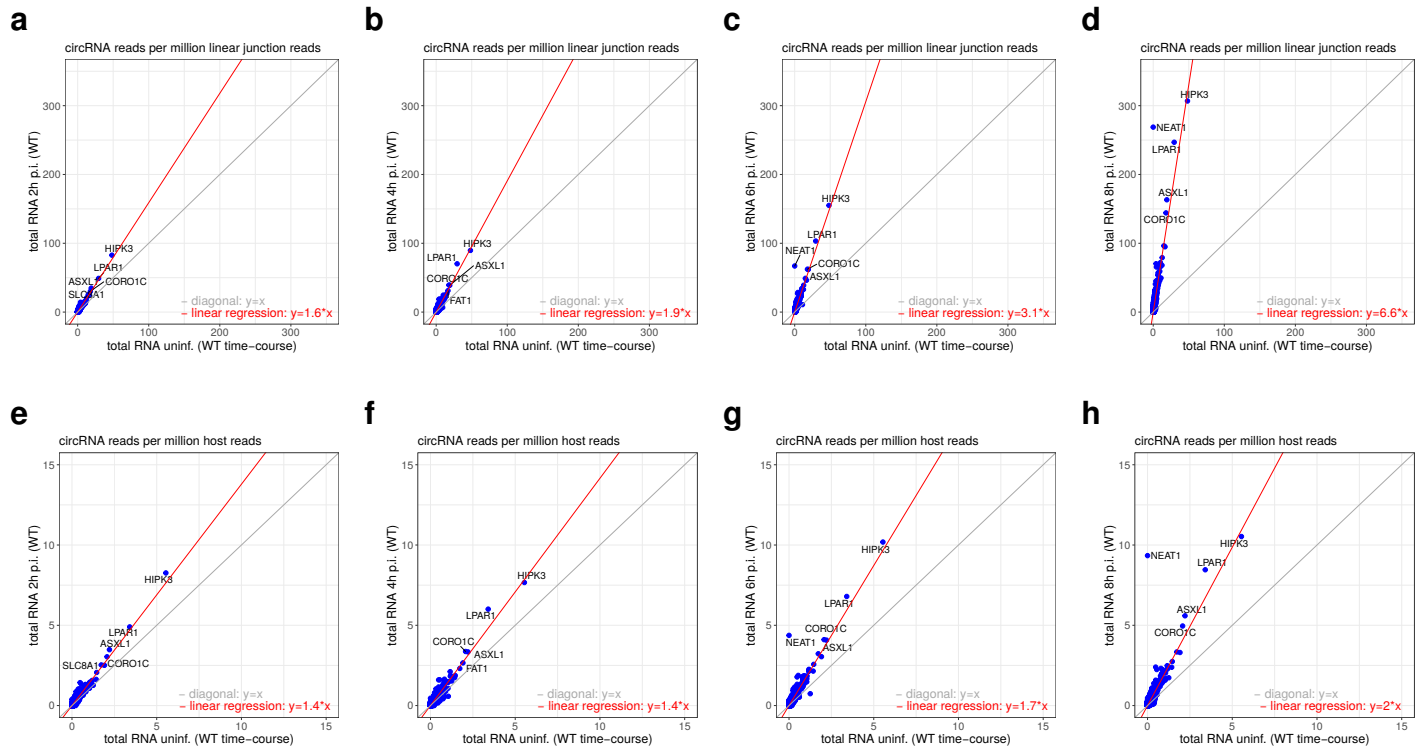

**S5 Fig**

Supplement: S5 Fig — Scatterplots comparing normalized circRNA counts (normalized to the number of linear junction reads (a-d) or to the total number of reads mapped to the host genome (e-f)) between mock and 2, 4, 6 and 8 h p.i. WT infection after trimming reads down to 76 nt. Linear regression analysis across all circRNAs (red line) was used to estimate the enrichment of circRNAs relative to linear mRNAs in HSV-1 infection compared to mock infection. The regression estimate for the enrichment is shown on the bottom right. The gray line indicates the diagonal, i.e., equal values on the x- and y-axis. The five most highly expressed circRNAs are marked by name. (PDF) [file pone.0276467.s005.pdf]

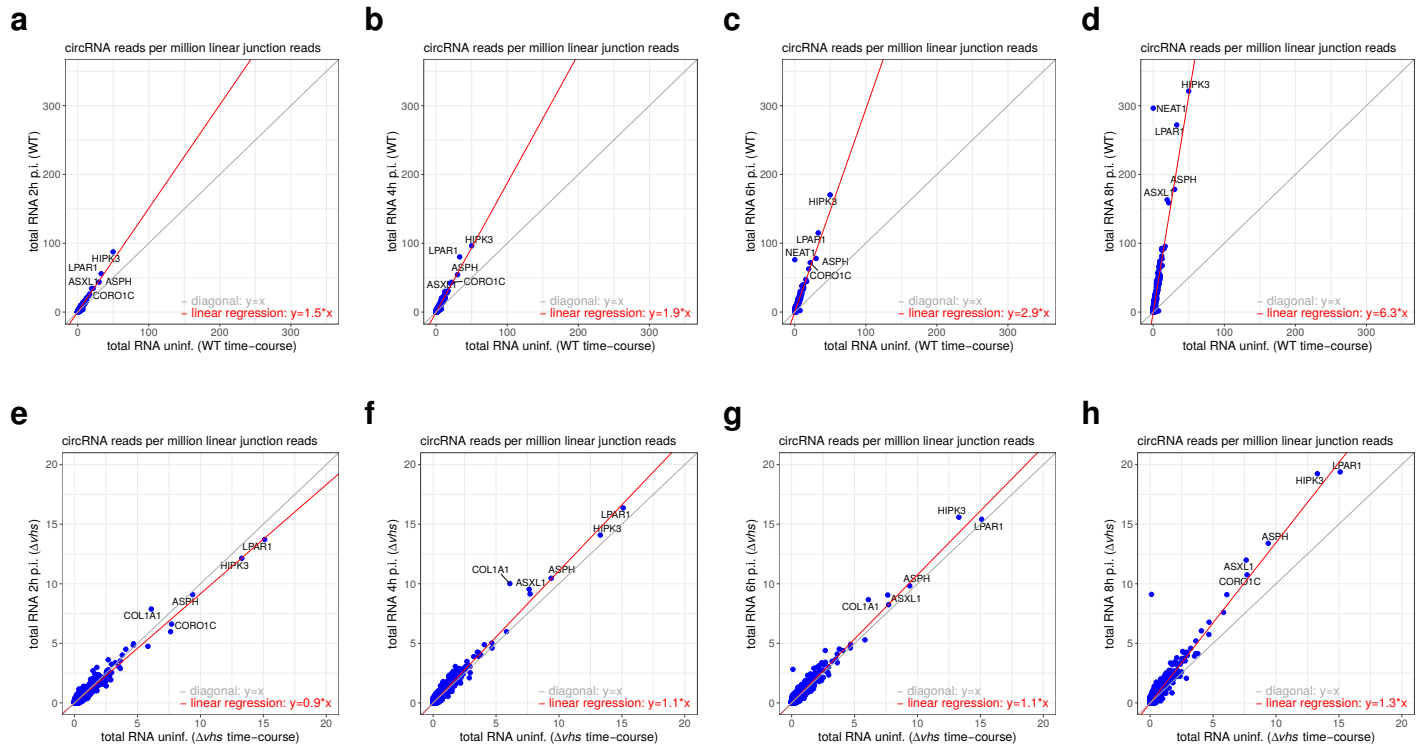

**S6 Fig**

Supplement: S6 Fig — Scatterplots comparing normalized circRNA counts (obtained with the alignment-based circRNA detection pipeline outlined in S4 Fig and normalized to the number of linear junction reads mapped to the host genome) between mock and 2, 4, 6 and 8 h p.i. WT infection (a-d) and between mock and 2, 4, 6 and 8 h p.i. Δvhs infection (e-h). Linear regression analysis across all circRNAs (red line) was used to estimate the enrichment of circRNAs relative to linear mRNAs in HSV-1 infection compared to mock infection. The regression estimate for the enrichment is shown on the bottom right. The gray line indicates the diagonal, i.e., equal values on the x- and y-axis. The five most highly expressed circRNAs are marked by name. (PDF) [file pone.0276467.s006.pdf]

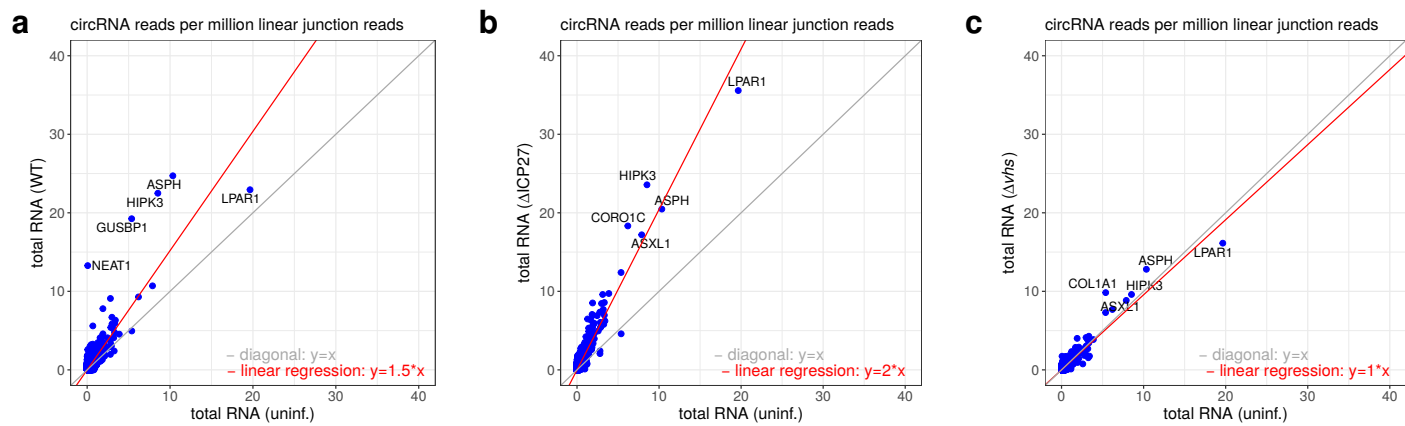

**S7 Fig**

Supplement: S7 Fig — Scatterplots comparing normalized circRNA counts (normalized to the number of linear junction reads mapped to the host genome) between mock and (a) WT, (b) ΔICP27 and (c) Δvhs infection. CircRNA read counts were obtained with the alignment-based circRNA detection pipeline outlined in S4 Fig. Linear regression analysis across all circRNAs (red line) was used to estimate the enrichment of circRNAs relative to linear mRNAs in HSV-1 infection compared to mock infection. The regression estimate for the enrichment is shown on the bottom right. The gray line indicates the diagonal, i.e., equal values on the x- and y-axis. The five most highly expressed circRNAs are marked by name. (PDF) [file pone.0276467.s007.pdf]

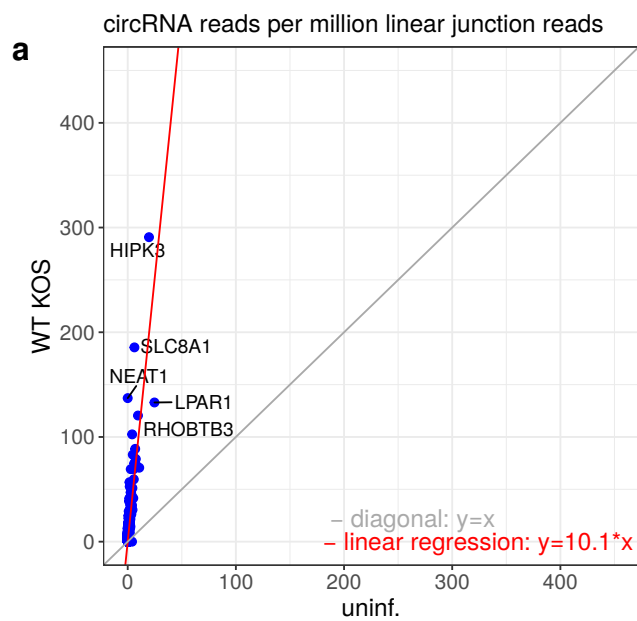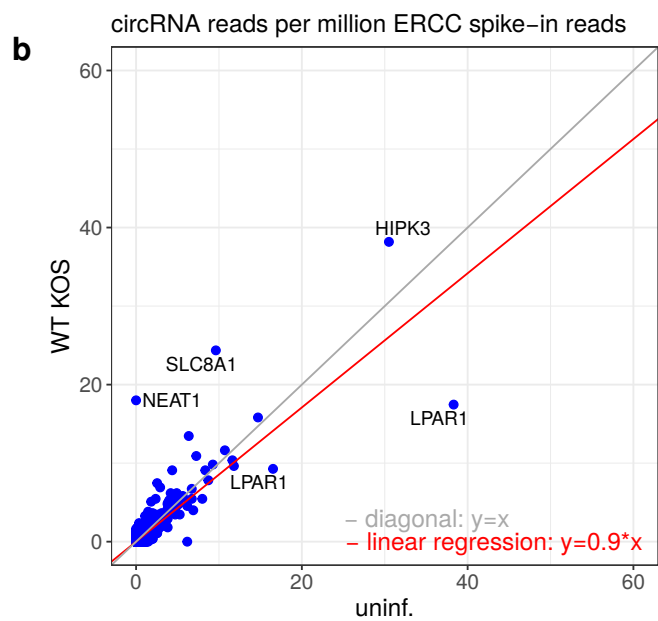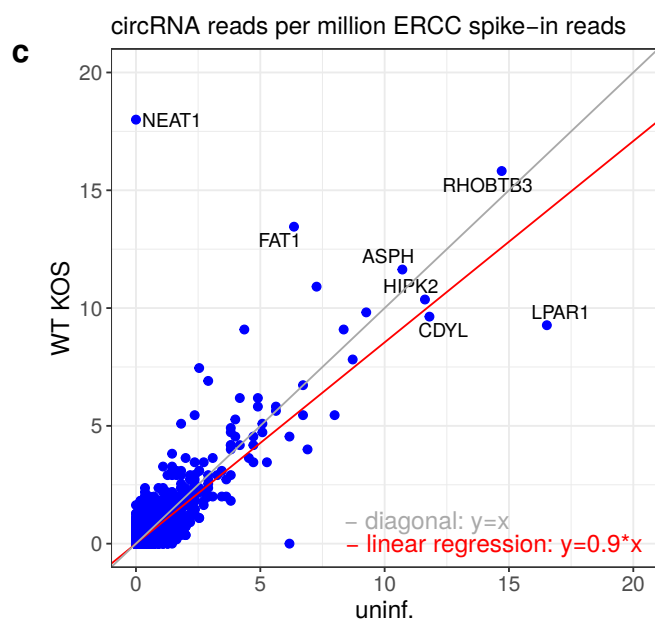

**S8 Fig**

Supplement: S8 Fig — (a-d) Scatterplots comparing circRNA counts obtained for mock and WT KOS infection from the study of Dremel et al. [53] normalized by either (a) the number of linear junction reads mapped to the host genome, or (b, c) the number of reads mapping to ERCC spike-in sequences. CircRNA read counts were obtained with the alignment-based circRNA detection pipeline outlined in S4 Fig. (c) shows the same data as (b) with axes restricted to the range of 0 to 20. Linear regression analysis across all circRNAs (red line) was used to estimate in (a) the enrichment of circRNAs relative to linear mRNAs in HSV-1 infection compared to mock infection and in (b, c) the change in absolute circRNA abundances. The regression estimate is shown on the bottom right. The gray line indicates the diagonal, i.e., equal values on the x- and y-axis. The five most highly expressed circRNAs are marked by name. Normalization to ERCC spike-in read counts shows no absolute increase for most circRNAs in HSV-1 infection compared to mock infection, indicating that circRNAs are enriched relative to linear transcripts due to a general reduction of linear mRNAs during HSV-1 infection. (PDF) [file pone.0276467.s008.pdf]

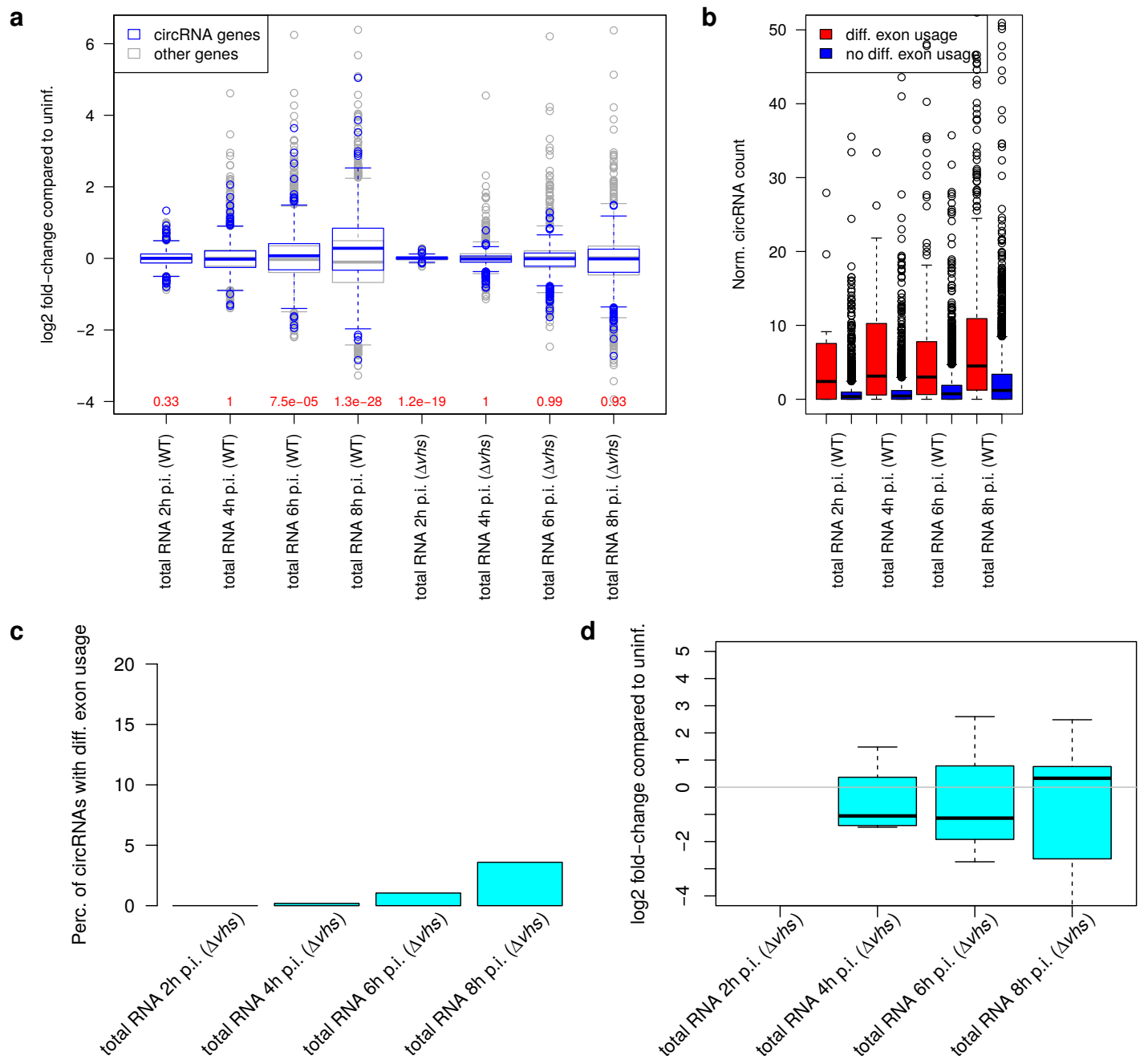

**S9 Fig**

Supplement: S9 Fig — (a) Boxplots showing the distribution of log2 fold-changes in gene expression between mock and 2, 4, 6 and 8 p.i. total RNA in WT and Δvhs infection for genes containing at least one circRNA (blue) or no circRNA (gray). log2 fold-changes were taken from our recent study [18] (see also methods). Wilcoxon rank sum tests were used to assess whether log2 fold-changes were significantly increased for circRNA genes compared to other genes at each time-point of infection (p-values shown in red at the bottom). (b) Boxplots showing the distribution of normalized circRNA counts for circRNAs with (red) and without (blue) differential exon usage for exons within the circRNA region at each time-point of WT infection. (c) Percentage of expressed circRNAs (= circRNA count >0 in uninfected cells) for which at least one exon within the genomic region of the circRNA shows differential exon usage for the corresponding gene (determined with DEXSeq, multiple testing adjusted p-value ≤ 0.005) for each time-point of Δvhs infection. (d) Boxplots showing the distribution of log2 fold-changes for exons located within circRNAs. For each circRNA, only the exon with the maximum absolute log2 fold-change is shown. (PDF) [file pone.0276467.s009.pdf]

no. circRNAs with higher expression:

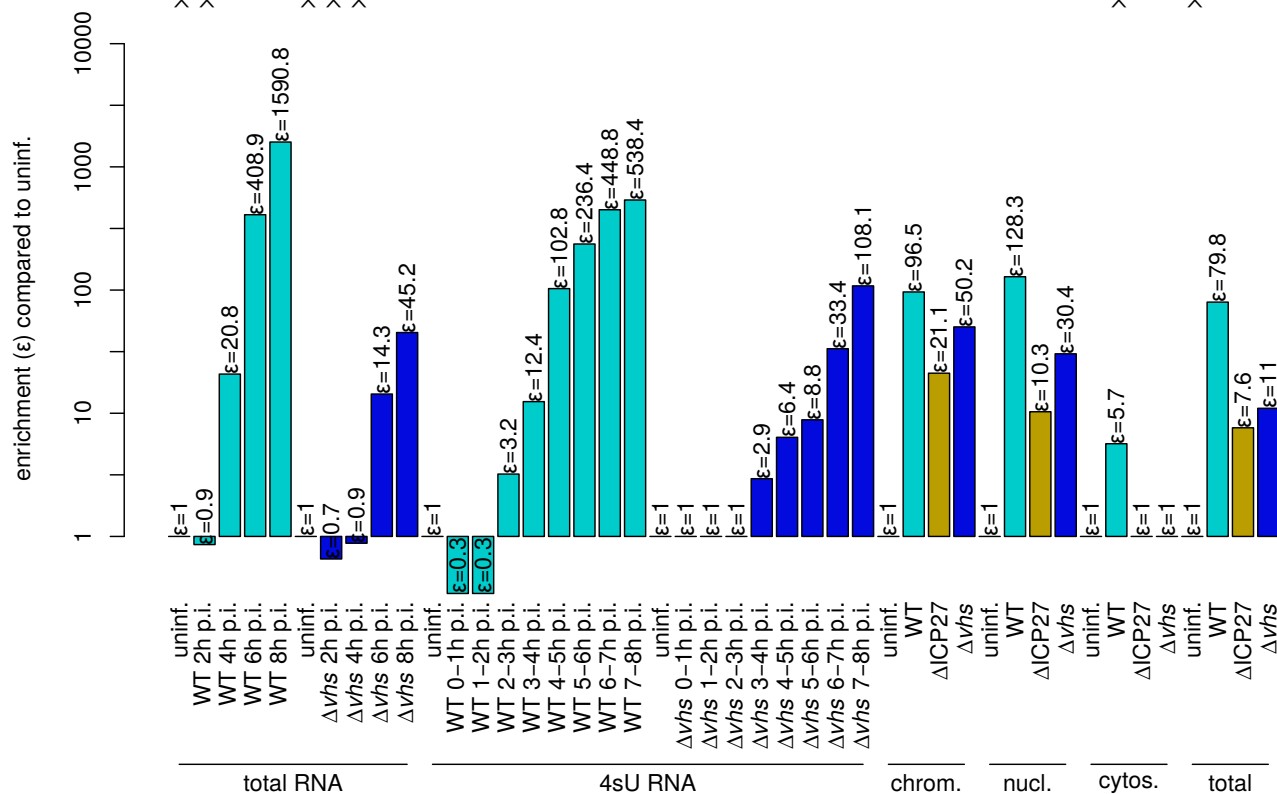

S10 Fig

Supplement: S10 Fig — Enrichment (: = ε) compared to mock infection was calculated as the ratio of normalized hsa_circ_0003812 circRNA counts between infected and uninfected cells. Since no or very few reads were obtained for the hsa_circ_0003812 NEAT1_2 circRNA in uninfected cells, a pseudocount of 0.1 was used to avoid division by zero. Numbers on top indicate the number of circRNAs identified in the corresponding condition with a higher normalized circRNA count than hsa_circ_0003812. NA indicates that no hsa_circ_0003812 circular junction reads were not found in the particular condition. (PDF) [file pone.0276467.s010.pdf]

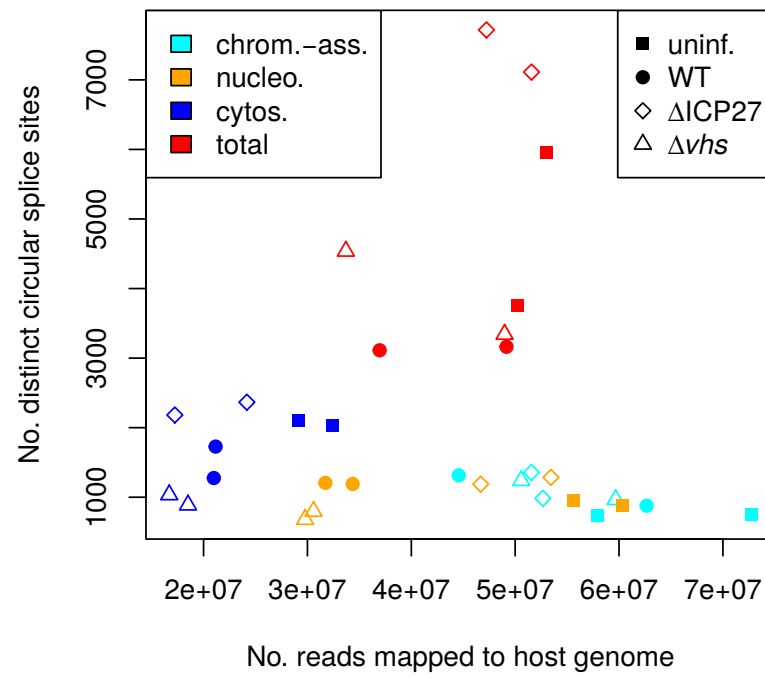

**S11 Fig**

Supplement: S11 Fig — (PDF) [file pone.0276467.s011.pdf]

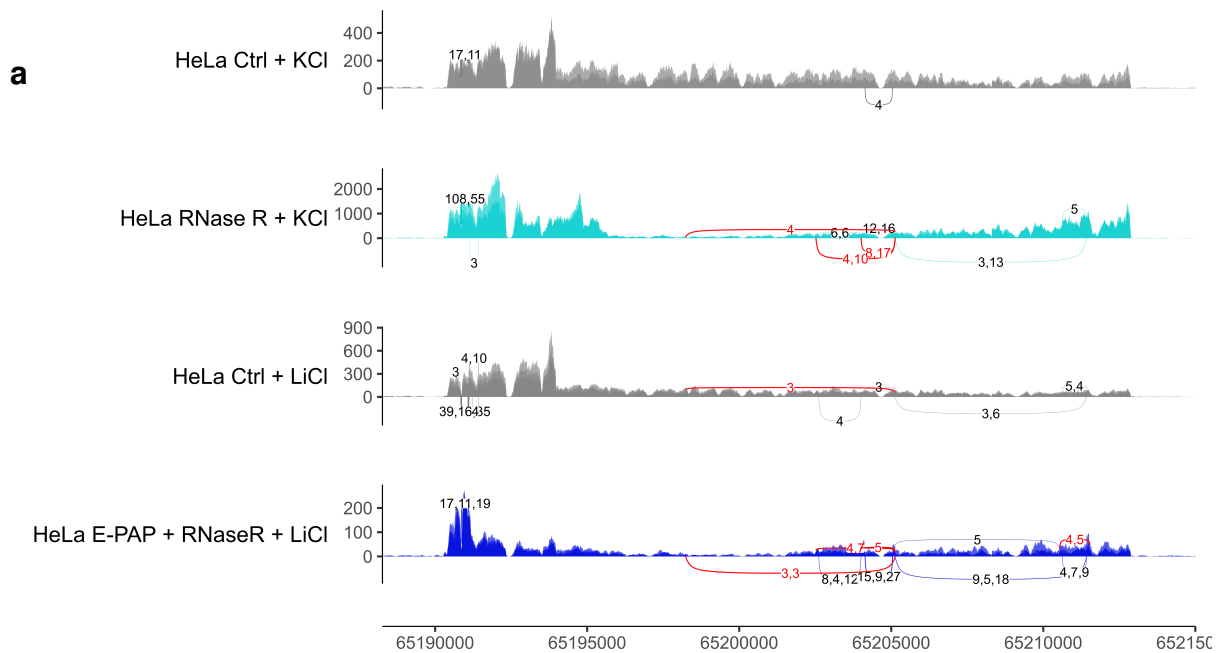

hsa\_circ\_0003812

NEAT1\_2

NEAT1\_1

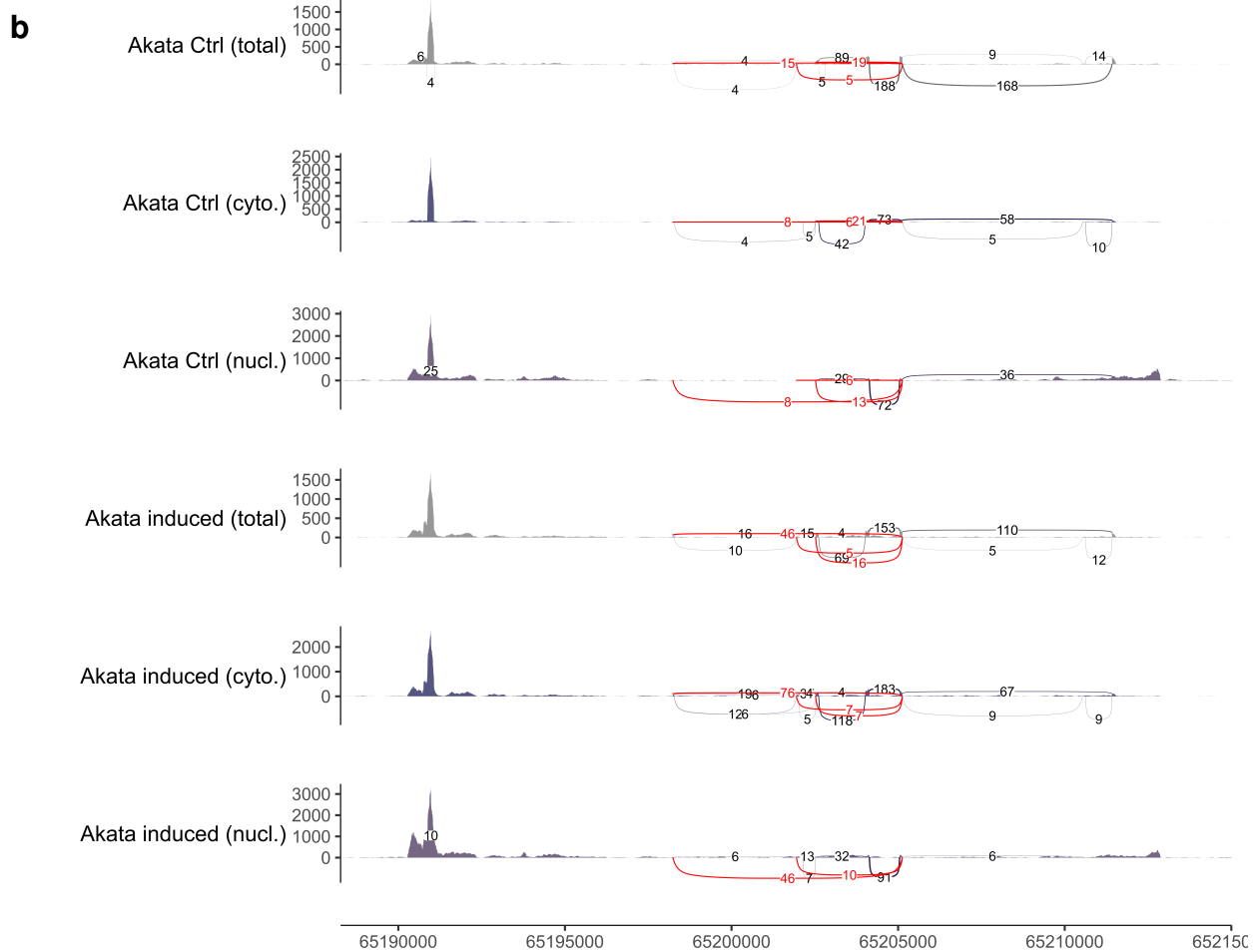

hsa\_circ\_0003812

NEAT1\_2

NEAT1\_1

**S12 Fig**

Supplement: S12 Fig — Sashimi plots show NEAT1 read coverage (overlay of replicates) and circular (red) and linear (same color as read coverage) splice junctions as arcs connecting acceptor and donor splice site in RNA-seq data from the studies by (a) Xiao and Wilusz [20] and (b) Ungerleider et al. [55] (for details see below). Number of junction reads are annotated to arcs separately for replicates. Junctions are only shown if at least 3 and 4 reads, respectively, align by at least 10 nt on both sides of the junction. Genomic coordinates of NEAT1 transcripts and the HSV-1-induced circRNA are shown at the bottom. (a) Xiao and Wilusz performed RNA-seq for HeLa cells with and without (= Ctrl) RNase R treatment using two different protocols. The first protocol is the standard approach used for enriching circRNAs and employs RNase R treatment with a KCl-containing buffer for 15 min (2 biological replicates). In the second protocol, RNA was treated by E-PAP followed by digestion with RNase R in a LiCl-containing buffer (3 biological replicates). (b) Ungerleider et al. performed RNase R treatment for total, cytoplasmic and nuclear RNA obtained from Akata cells without (= Ctrl) and with induction of EBV activation. (a, b) NEAT1_2 is partially resistant to RNase R treatment due to the stabilizing triple helical structure at its 3’end, thus even after RNase R treatment reads from the linear NEAT1_2 transcript, in particular its 3’end, are obtained for total RNA from HeLa cells and nuclear RNA from Akata cells. For total and cytoplasmic RNA from Akata cells (both induced and noninduced), depletion of the linear NEAT1_2 transcript was successful. (PDF) [file pone.0276467.s012.pdf]

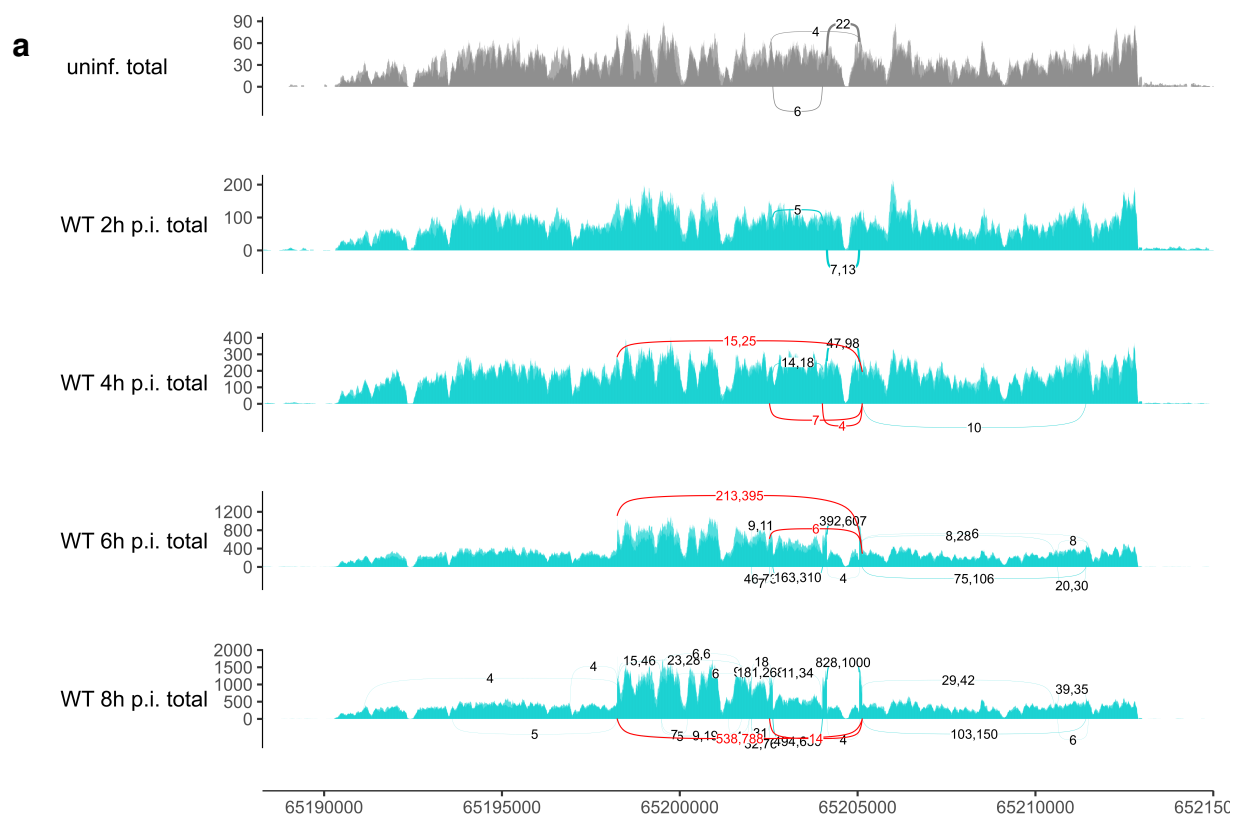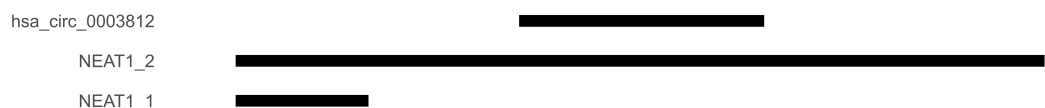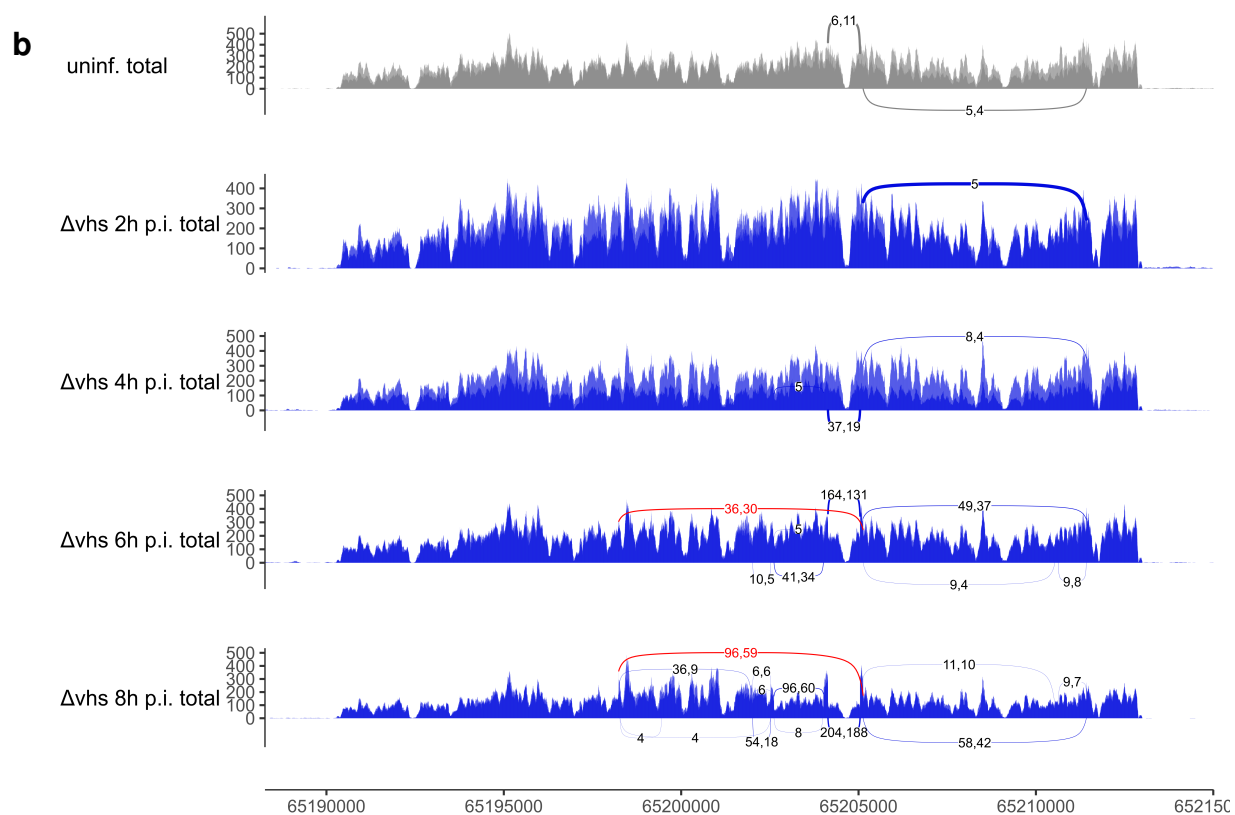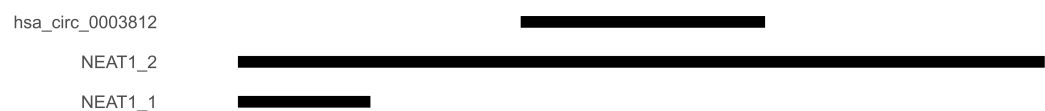

**S13 Fig**

Supplement: S13 Fig — For an explanation of sashimi plots see caption to Fig 3. Circular splice junctions are marked in red, linear splice junctions in the same color as read coverage. (PDF) [file pone.0276467.s013.pdf]

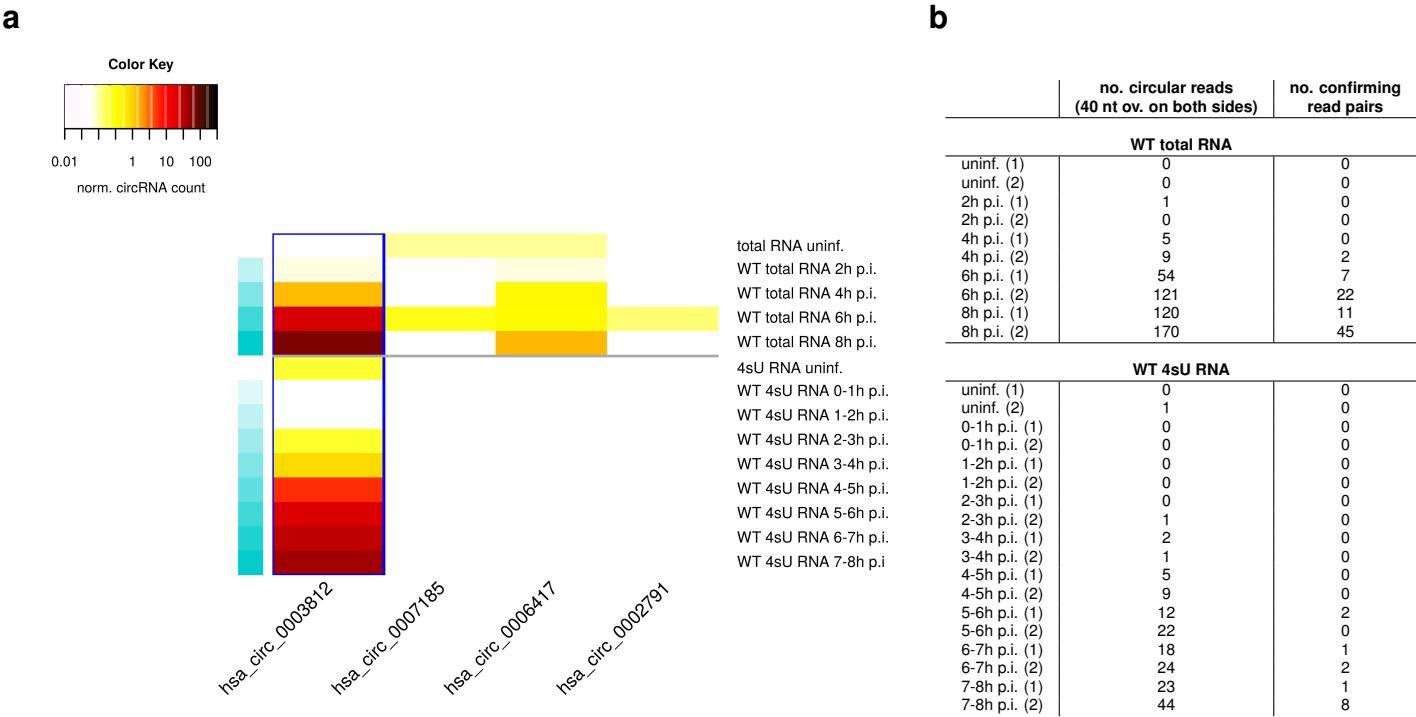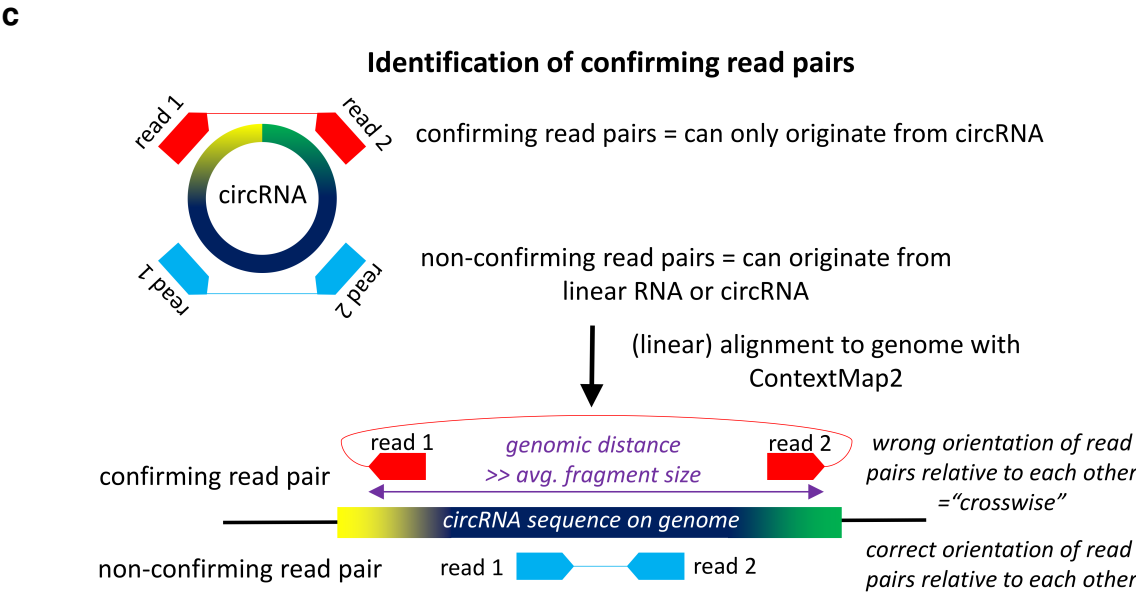

S14 Fig

Supplement: S14 Fig — (a) Heatmap of normalized circRNA counts (normalized to the number of linear junction reads mapped to the host genome) for all NEAT1_2 circRNAs in total and 4sU-RNA time-courses of WT infection identified with the alignment-based approach with a minimum overlap of at least 40 nt on either side of the circular junction. Columns represent individual circRNAs, which are ordered according to their genomic coordinates from the most 5’ to the most 3’. The hsa_circ_0003812 NEAT1_2 circRNA is marked by a blue rectangle. (b) Read counts for circular junction reads for hsa_circ_0003812 identified with the alignment-based approach with a minimum overlap of at least 40 nt on either side of the circular junction (i.e., raw read counts used for calculating normalized circRNA counts for (a)) and read counts for confirming read pairs for hsa_circ_0003812 identified with the approach outlined in (c). Numbers are shown separately for both replicates of the total and 4sU RNA time-courses of WT infection. (c) Definition and detection of confirming read pairs for circRNAs. For further details see methods. (PDF) [file pone.0276467.s014.pdf]

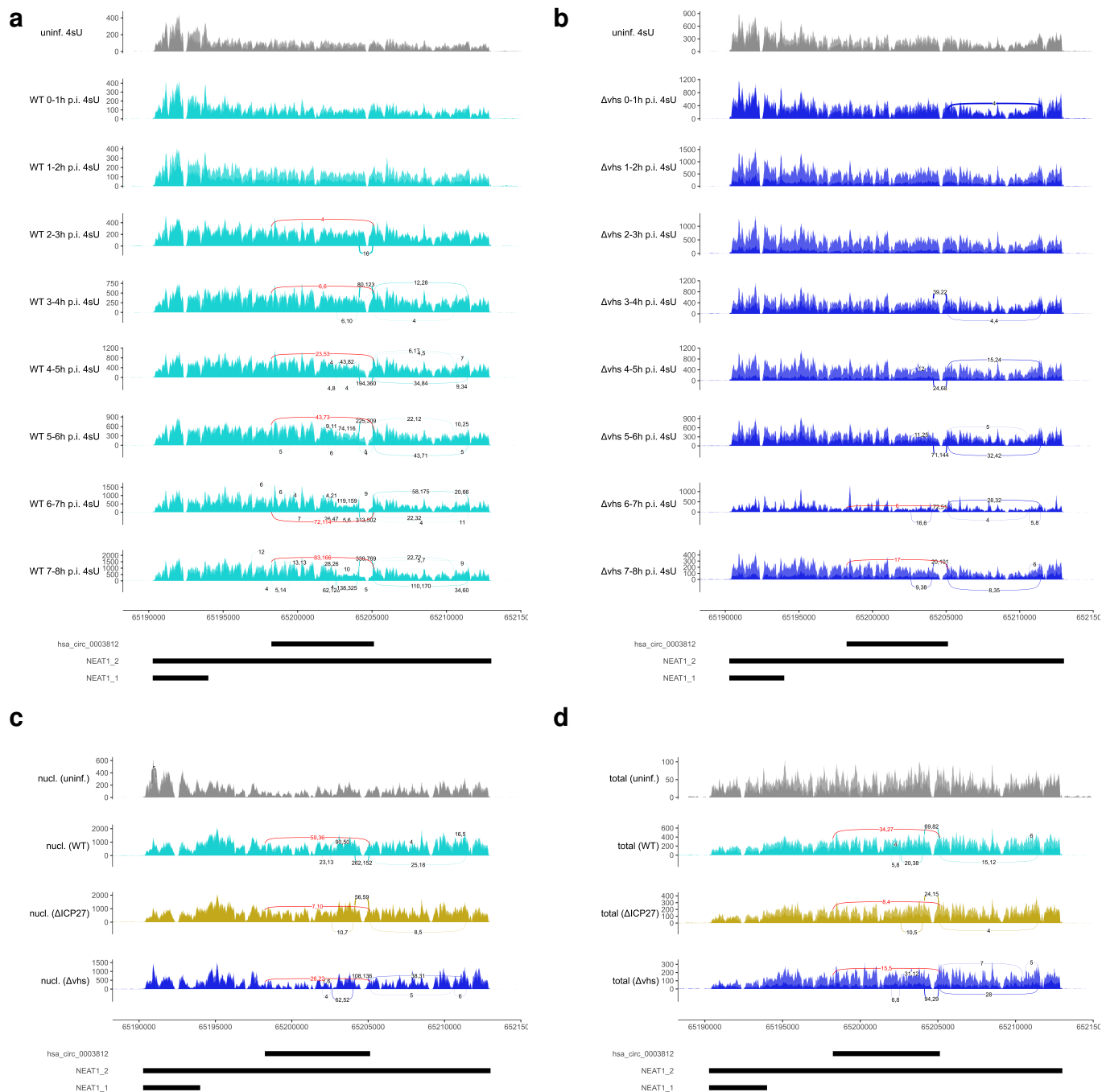

S15 Fig

Supplement: S15 Fig — Sashimi plots for 4sU-RNA time-courses of (a) WT and (b) Δvhs infection and (c) nucleoplasmic and (d) total RNA from the subcellular fractions experiment for WT, Δvhs and ΔICP27 infection. For an explanation of sashimi plots see caption to Fig 3. Circular splice junctions are marked in red, linear splice junctions in the same color as read coverage. (PDF) [file pone.0276467.s015.pdf]

## donor splice site

## acceptor splice site

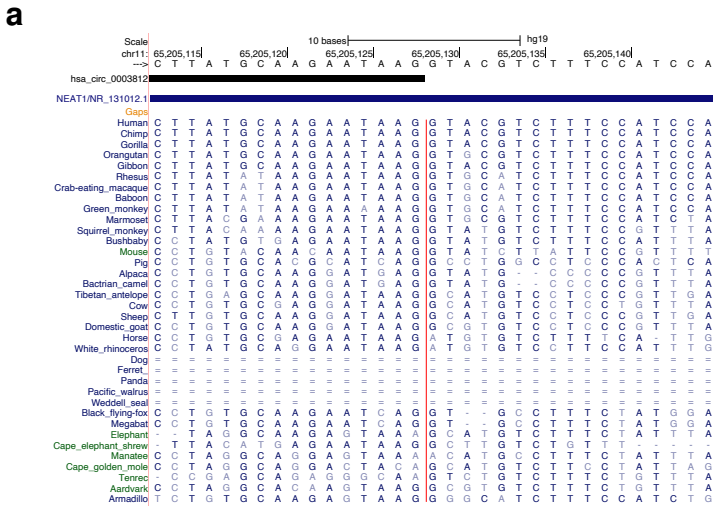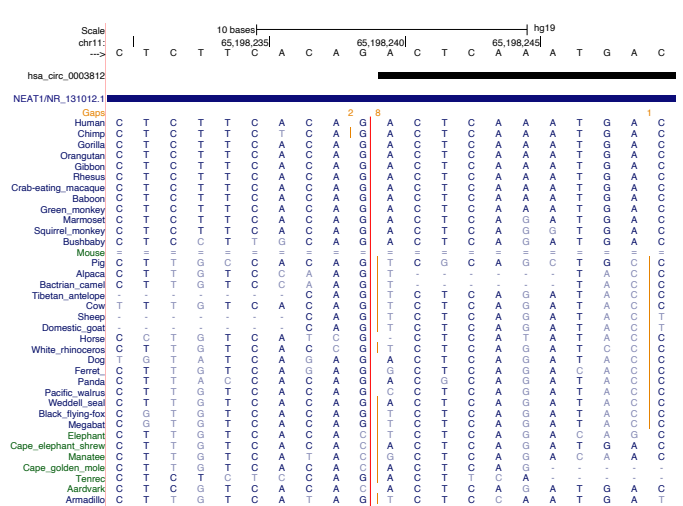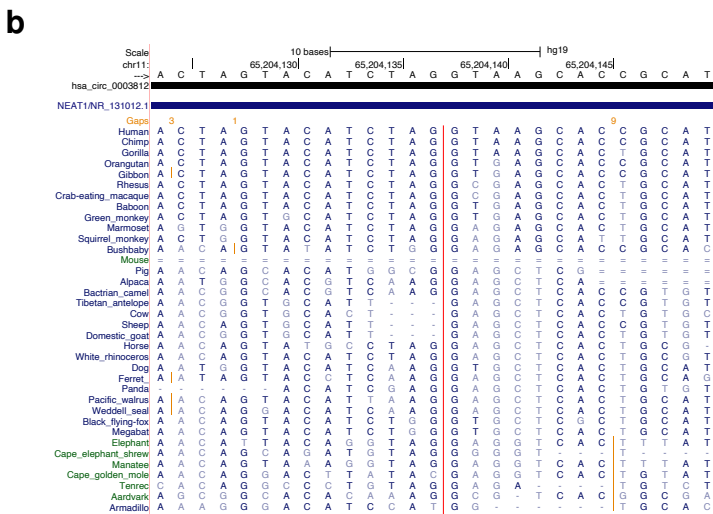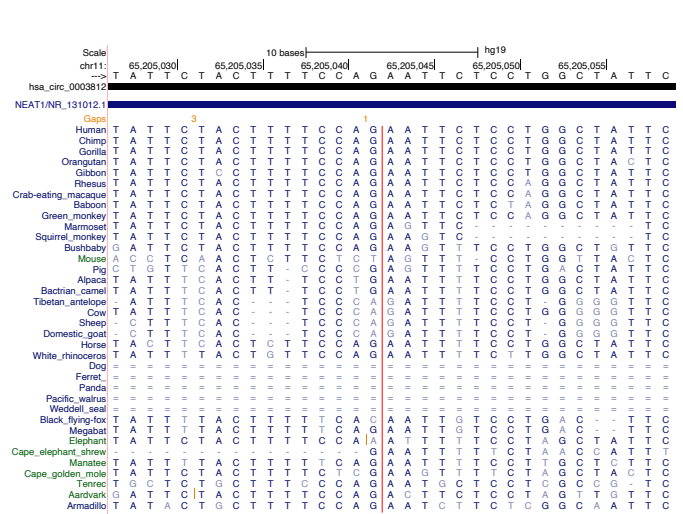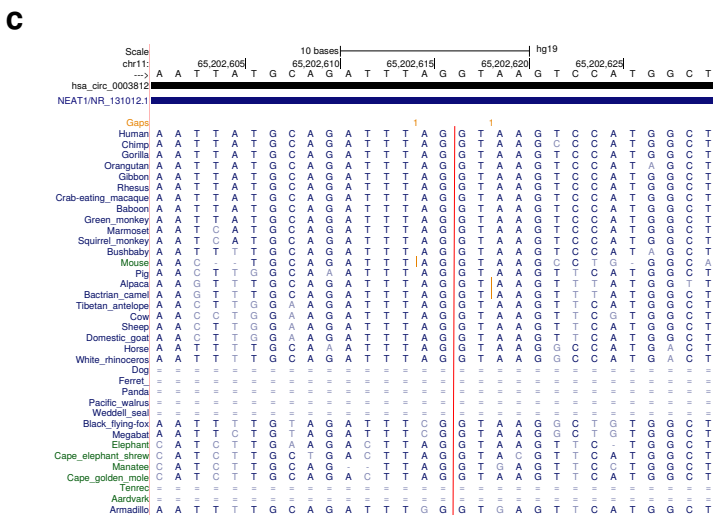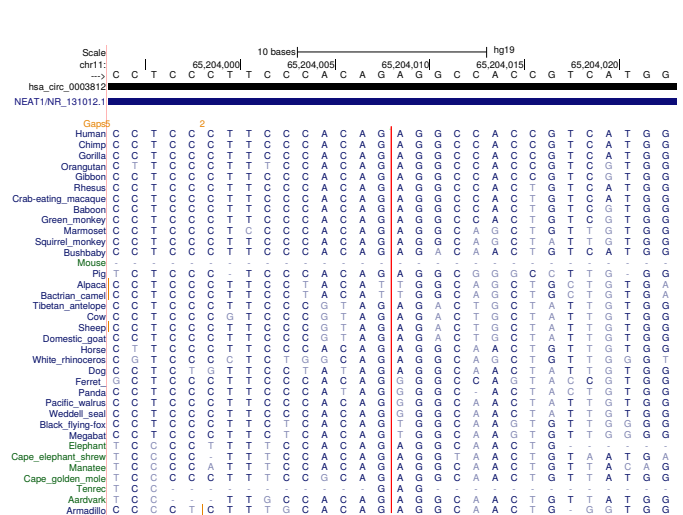

S16 Fig

Supplement: S16 Fig — Conservation of splice sites for (a) the hsa_circ_0003812 circular junction and (b-c) the two most frequent linear NEAT1_2 splice junctions (numbered (1) and (2) in Fig 3E). The left columns show the donor splice site (canonical GT signal at the intron 5’ end) and the right column the acceptor splice site (canonical AG at the intron 3’end), with the red vertical lines marking the exon-intron boundaries. Please note that for the circular junction in (a), the donor splice site is downstream of the acceptor splice site on the genome. Genome alignments against the human genome (hg19) were obtained from the UCSC genome browser. (PDF) [file pone.0276467.s016.pdf]

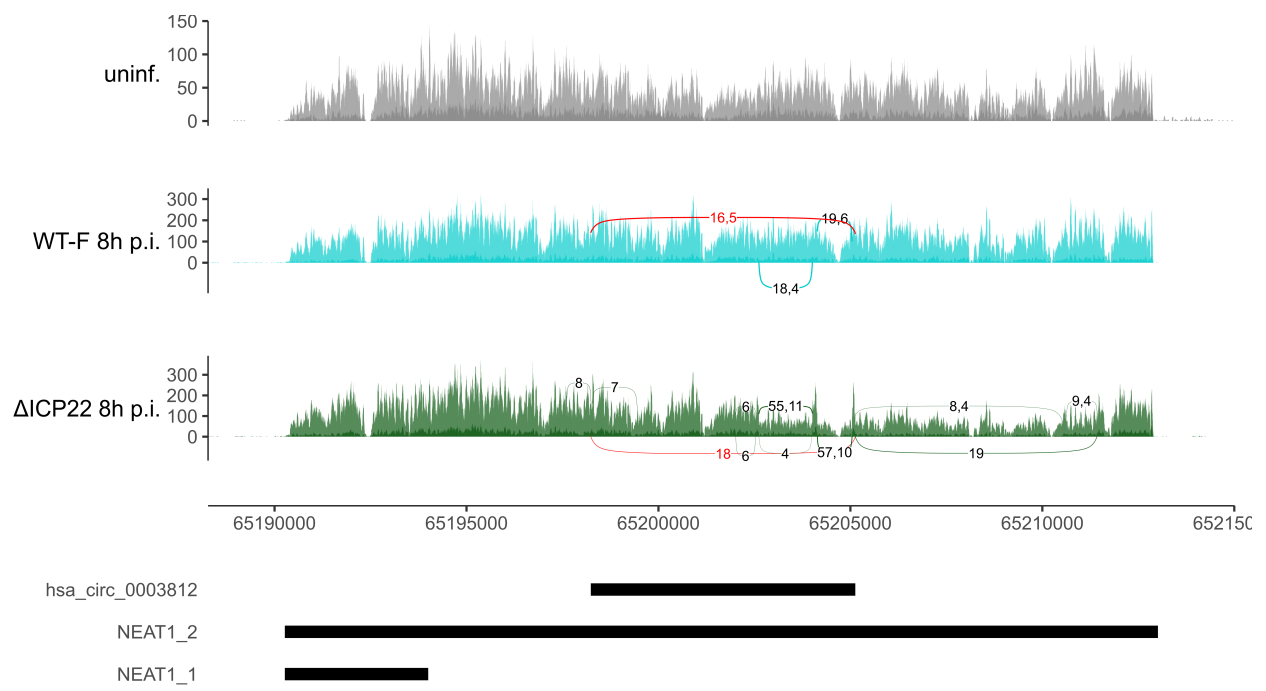

S17 Fig

Supplement: S17 Fig — For an explanation of sashimi plots see caption to Fig 3. Circular splice junctions are marked in red, linear splice junctions in the same color as read coverage. (PDF) [file pone.0276467.s017.pdf]

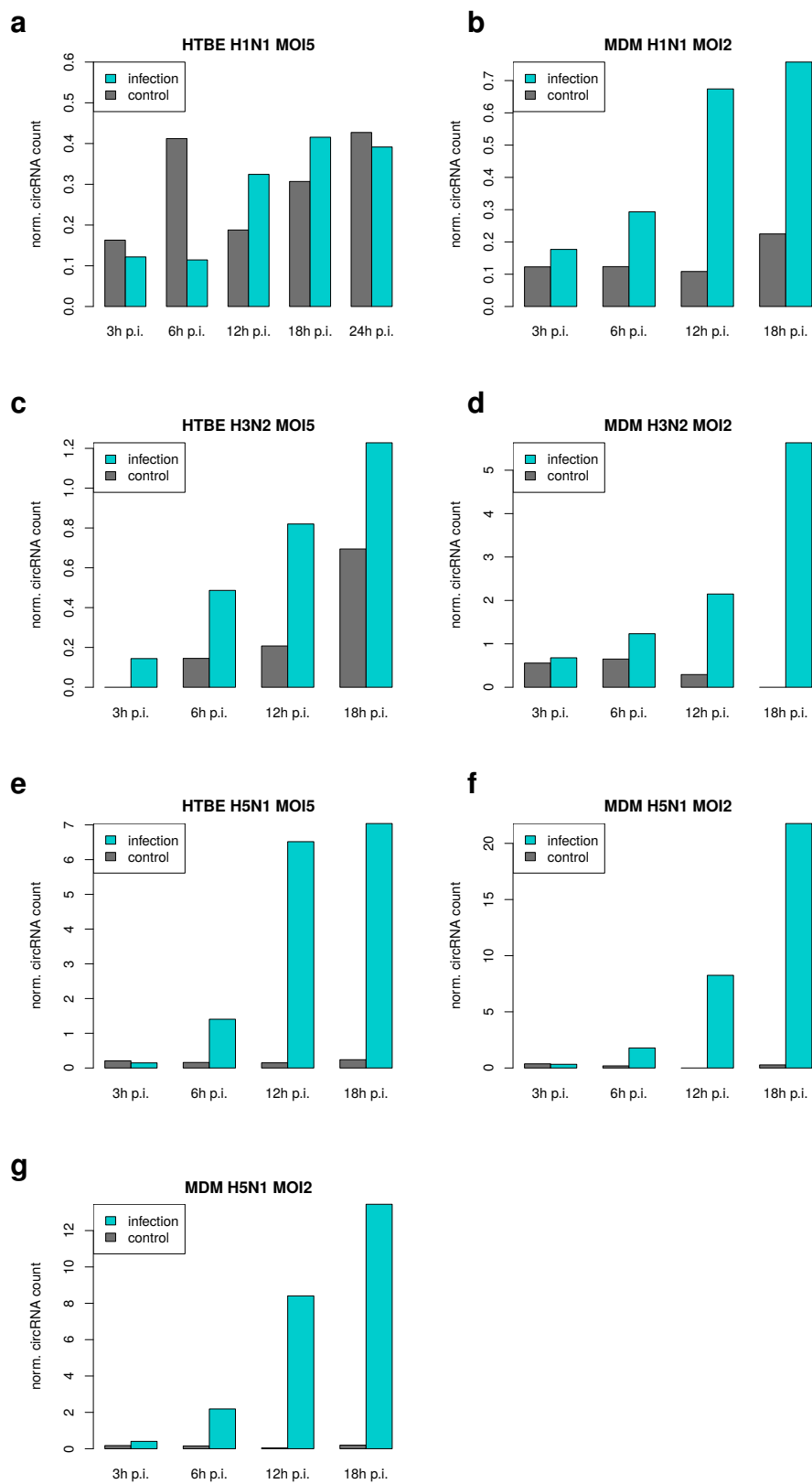

S18 Fig

Supplement: S18 Fig — Results are shown separately for H1N1, H3N2 and H5N1 infection and HTBE and MDM cells. (PDF) [file pone.0276467.s018.pdf]

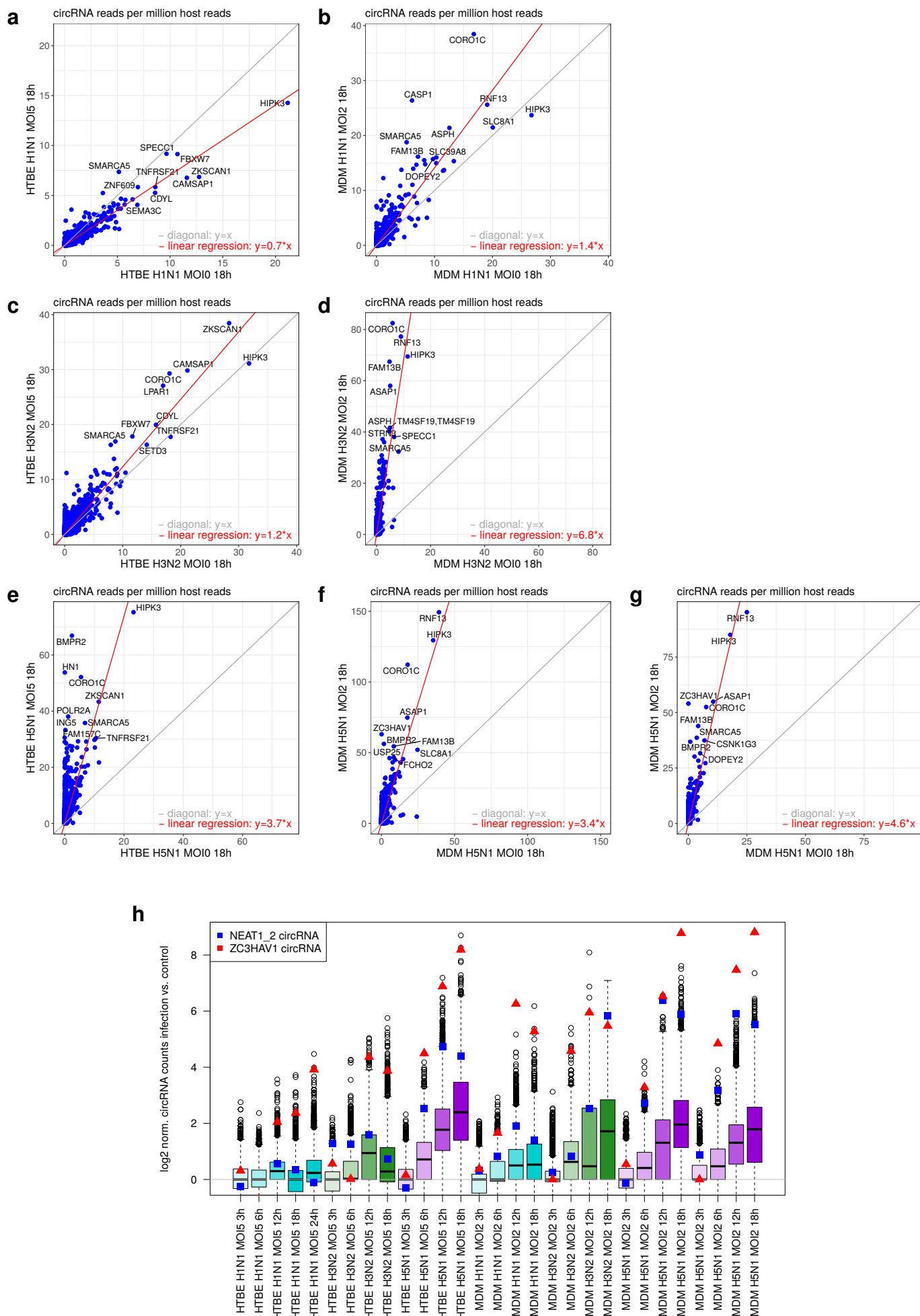

**S19 Fig**

Supplement: S19 Fig — (a-g) Scatterplots comparing normalized circRNA counts (normalized to the number of linear junction reads mapped to the host genome) for IAV infections at 18 h p.i. against time-matched controls. Linear regression analysis across all circRNAs (red line) was used to estimate the enrichment of circRNAs relative to linear mRNAs in IAV infection compared to time-matched controls. The regression estimate for the enrichment is shown on the bottom right. The gray line indicates the diagonal, i.e., equal values on the x- and y-axis. The most highly expressed circRNAs are marked by name. (h) Boxplots showing the distribution of log2 fold-changes between normalized circRNA counts in infection compared to time-matched controls for 7077 well-expressed circRNAs (normalized circRNA count >1 in at least one condition). A pseudocount of 0.1 was used to avoid division by zero. IAV strain and time-points of infection are color-coded (cyan = H1N1, green = H3N2, purple = H5N1, darker colors indicate later time-points). Values for the hsa_circ_0003812 NEAT1_2 circRNA and the strongly enriched ZC3HAV1 circRNA are shown as blue squares and red triangles, respectively. (PDF) [file pone.0276467.s019.pdf]

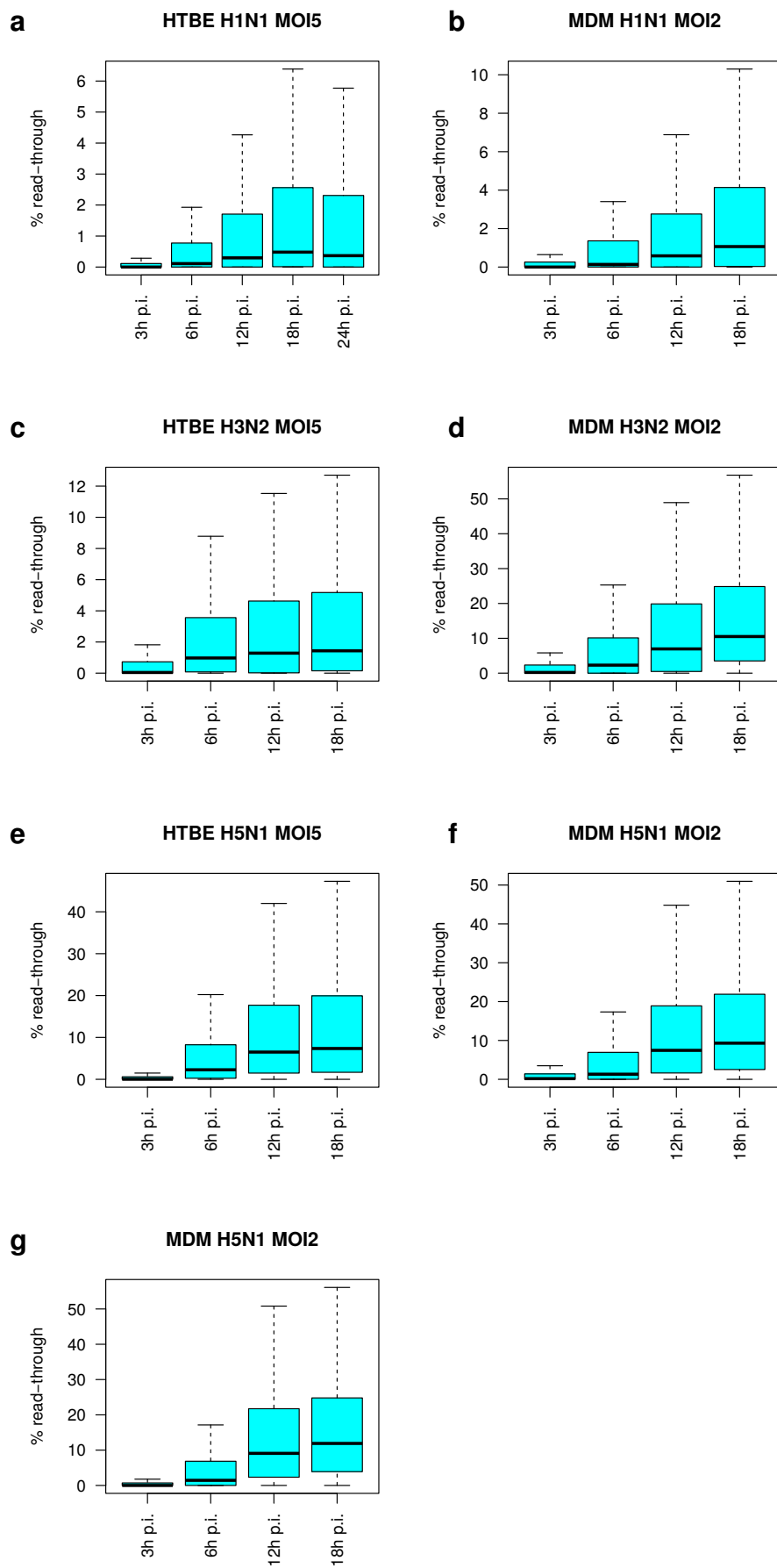

S20 Fig

Supplement: S20 Fig — Read-through was calculated as previously described in [22] (see also methods). (PDF) [file pone.0276467.s020.pdf]

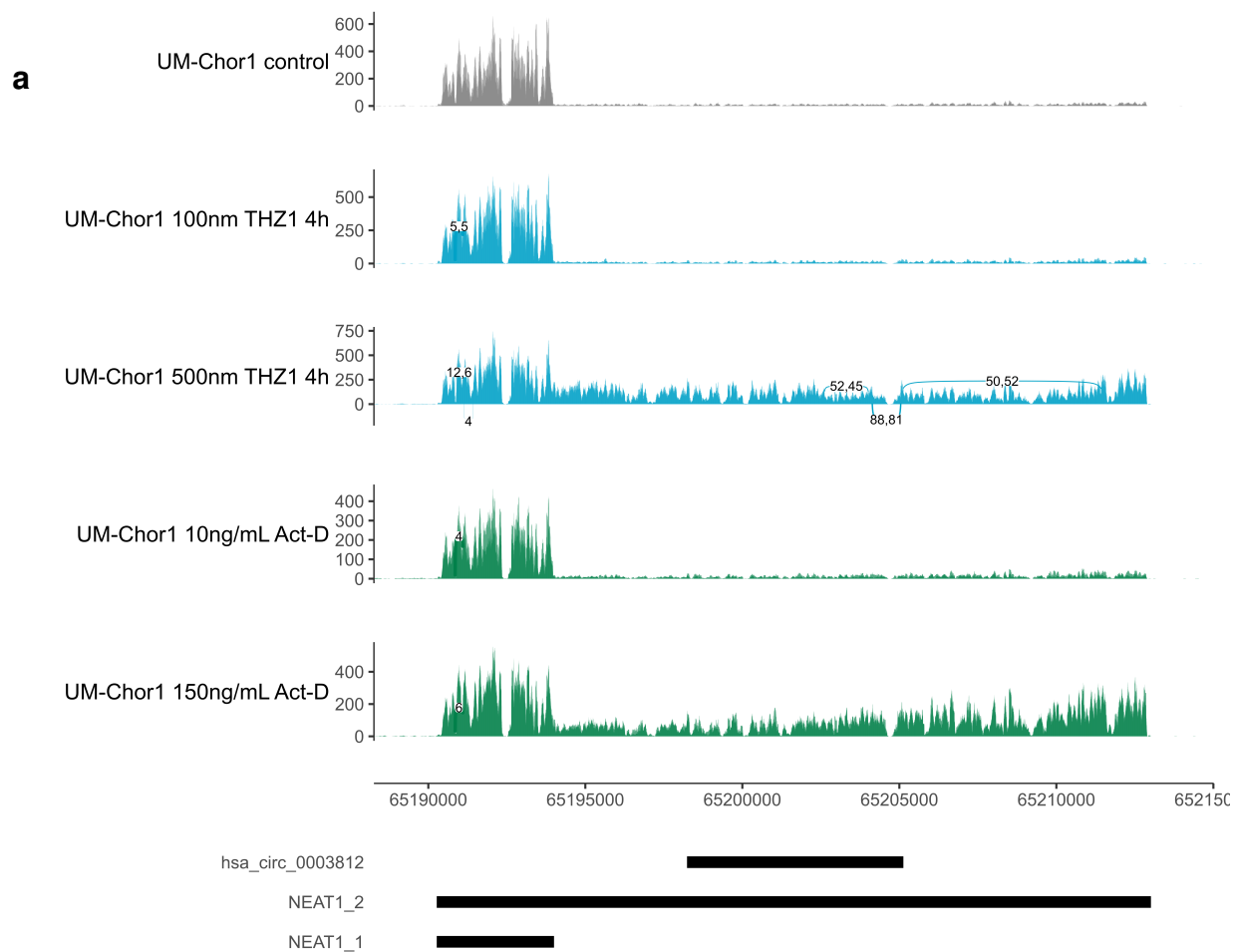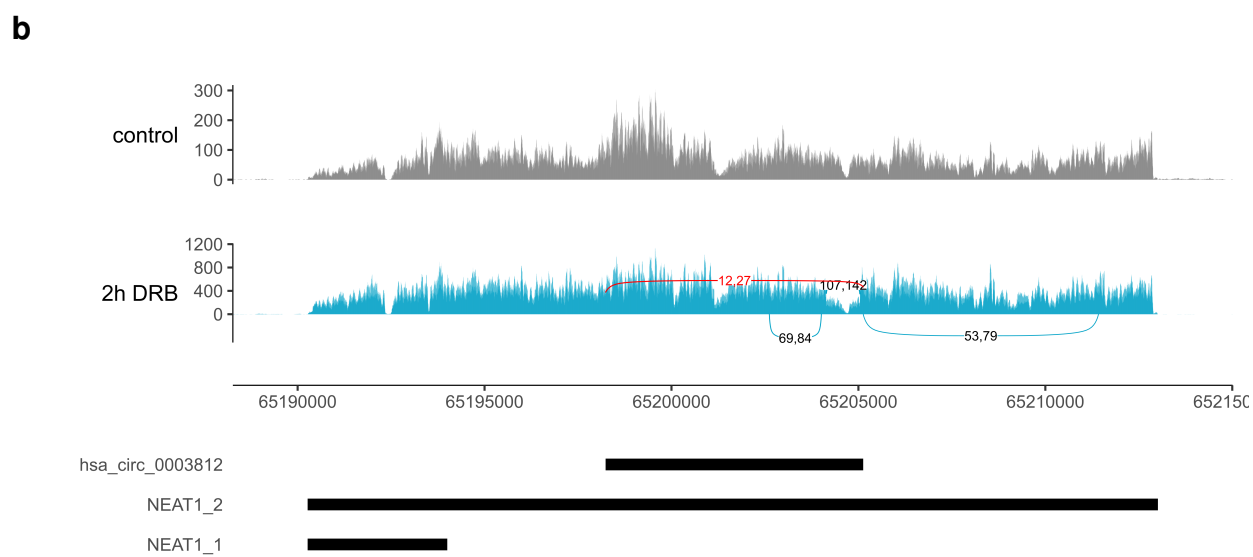

**S22 Fig**

Supplement: S22 Fig — Sashimi plots showing read coverage and splice junctions upon (a) control, THZ1 or Act-D treatment in different concentrations in UM-Chor1 cells (2 replicates each) and (b) control or DRB treatment in HEK293 cells (2 replicates each). For an explanation of sashimi plots see caption to Fig 3. No circular splice junctions were found, thus only linear splice junctions are shown. (PDF) [file pone.0276467.s022.pdf]
