# Supplementary material for: HSV-1 and influenza infection induce linear and circular splicing of the long NEAT1 isoform
Source: PLoS One. 2022 Oct 24;17(10):e0276467. doi: 10.1371/journal.pone.0276467 (PMC9591066; doi:10.1371/journal.pone.0276467)
Supplement: S21 Fig — (a) Overview of number of projects and samples that were identified with the Snaptron search in recount3 for the four most frequent NEAT1_2 linear splice junctions induced in HSV-1 infection. The left-most column indicates regular expressions that were searched in the project and sample descriptions provided by recount3 using grep in R (case insensitive). The table shows only projects and samples for which at least one of the four most frequent NEAT1_2 linear splice junctions is covered by ≥ 10 reads. (b) Sashimi plots as in Fig 3 for two RNA-seq experiments (3 replicates each) in erythrocytes [70] and platelets [69]. For an explanation of sashimi plots, see caption to Fig 3. Circular splice junctions are marked in red, linear splice junctions in black. (PDF) [file pone.0276467.s021.pdf]

a

	no. projects	no. samples
**all results**	909	6391
**pattern in description**		
(HSV-1|herpes simplex virus)	7	36
(influenza|H1N1|H3N2|H5N1)	7	63
(circRNA|circular RNA)	62	344
RNase R	21	136
blood	179	2417
platelet	13	65
erythrocyt	3	27
monocyt	60	541
leu[ck]ocyt	13	85
lymphocyt	22	137
macrophag	42	333
leukem	53	356
PBMC	34	323
K562	21	497
(CDK7|THZ1)	9	26

b

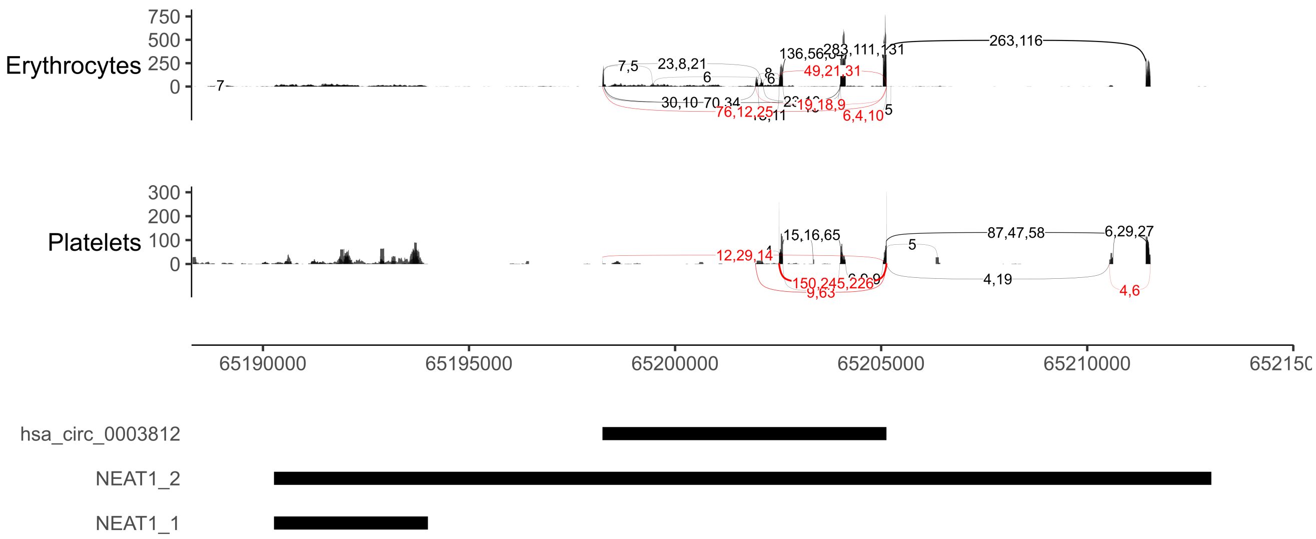

S21 Fig
