# Supplementary material for: HSV-1 and influenza infection induce linear and circular splicing of the long NEAT1 isoform
Source: PLoS One. 2022 Oct 24;17(10):e0276467. doi: 10.1371/journal.pone.0276467 (PMC9591066; doi:10.1371/journal.pone.0276467)
Supplement: Attachment — Submitted filename: Response_to_reviewers.pdf [file pone.0276467.s023.pdf]

Response to Reviewers' comments:

We would like to thank all reviewers for their helpful comments. We believe that addressing these comments has substantially strengthened the manuscript and clarified our analysis.

Reviewer #1:

In manuscript entitled "HSV-1 and influenza infection induce linear and circular splicing of the long NEAT1 isoform" authors combine multiple bioinformatics approaches to highlights potential role of NEAT1_2 in splicing and CDK7 in HSV-1 and/or IAV infection. Although the project design and manuscript preparations generally (see major point below) followed coherence with existing literature and adds valid contributions to the field, the manuscripts requires significant revisions and polishing in order to be published.

The major concern is to provide a more clear-cut definition of the observed alterations. The authors refer to "increase" or "decrease" or "induce" in certain circRNAs and common linear RNAs. Only from the design of the method, which is vaguely described, it may be assumed they are referring to relative alterations in library-size-normalised comparisons. Is this so? If yes, it need to be made very clear, as not all claims made in the manuscript would be supported by these data. Library-normalised alterations are not at all indicative neither of extent nor direction of absolute (actual concentration/abundance) alterations. Further, when assessed during events that trigger global transcriptme re-shaping, comparisons between conditions with regular approaches based on the assumption that only a small fraction of the transcriptome changes become unreliable or invalid. In essence, I think the authors need to provide additional/orthogonal proof to support the claims, such as FISH detection of the circRNA in situ, or its absolute quantification by other meas. The alternative is to substantially downplay the claims.

As the reviewer correctly noted, we were not precise in our wording by using the terms "increase", "induce" or "accumulation" both for (i) a relative enrichment of most circRNAs compared to linear RNAs due to degradation of these linear RNAs mediated by viral proteins (*vhs* in case of HSV-1 infection, PA-X and RNA-dependent RNA polymerase in case of influenza A infection) and (ii) for an absolute increase/induction in case of the NEAT1_2 circRNA and linear splicing events. We have revised the manuscript thoroughly to use a more careful wording using the term "enrichment" for (i) and "increase/induction" only for (ii). We also clarified our initial hypothesis in the introduction, i.e., that *vhs*-mediated degradation of linear RNAs that spares circRNAs would lead to an enrichment of circRNAs similar to, but likely less pronounced than RNase R treatment. As the reviewer pointed out, this confounds any differential circRNA analysis based on library normalizing as used e.g. in the previous study of circRNAs in HSV-1 infection by Shi *et al.*

We also clarified the description and the purpose of the normalization we used. CircRNA read counts were normalized to the total number of linearly spliced reads from all protein-coding genes in each sample not only the "parent" genes from which corresponding circRNAs originated (see also response to comment below). The purpose of this was to quantify the relative enrichment or circRNAs compared to all linear transcripts to determine the impact of virus-mediated decay of linear transcripts.

In addition, we included a new analysis of a recent total RNA-seq study on infection with the HSV-1 KOS strain, which used ERCC spike-ins to allow absolute quantification of transcript levels (Dremel *et al*., Nature Communications, 2022). This showed that absolute levels of almost all circRNAs are not fundamentally changed by HSV-1 infection (with the notable exception of the NEAT1_2 circRNA, which is indeed increased in absolute levels). Thus, *vhs*-mediated decay of linear transcripts explains the enrichment of circRNAs in HSV-1 infection.

Finally, we revised the manuscript to better present the evidence for an actual increase in NEAT1_2 circular and linear splicing in both HSV-1 and influenza A infection. Notably, since both linear and circular NEAT1_2 splicing is essentially absent in uninfected fibroblasts, any type of normalization would show an induction, however the ERCC spike-in normalized data from the Dremel *et al*. study clearly confirm an absolute increase in NEAT1_2 circular and linear splicing during HSV-1 infection.

If I understand the statement in lines 154-157, the circRNA reads were normalised to the read counts of the genes producing them. All circRNA-producing genes or specific to the circRNA type? Remains unclear. In any case, this seems as a potentially dangerous approach as outlined in the paragraph above.

As noted above, we clarified in the manuscript that circRNA read counts were normalized to the total number of linear junction reads of *all* human protein-coding genes (not only the "parent" genes from which circRNAs originate) to determine enrichment of circRNAs relative to linear transcripts. The reason we did not normalize only against reads from "parent" genes is that reads that map linearly to splice junctions within circRNA regions could originate from either linear or circular transcripts. Excluding those reads would result in low numbers of reads for normalization, leading to very noisy estimates of enrichment, in particular for HSV-1 infection where linear transcripts are expected to be lost. Including those reads, however, would lead to under-estimation of circRNA enrichment. Since only a relatively small fraction of genes harbor circRNAs, use of junction reads from all genes circumvents this issue. Moreover, there are circRNAs without corresponding linear transcripts, such as CDR1as, one of the few circRNAs with a well-described function. This circRNA would otherwise have to be excluded from our analysis.

More minor points that are nonetheless important to address re summarised below.

Language needs edits and editing. Already in the abstract it is stated (lines 17-18) "vhs-mediated degradation of mRNAs leads to an accumulation of circular 18RNAs (circRNAs) relative to linear mRNAs". Perhaps what is meant is "preferential accumulation of circRNAs versus linear RNA forms"?

We changed this sentence to: "Here, we show that *vhs*-mediated degradation of linear mRNAs leads to an enrichment of circRNAs relative to linear mRNAs during HSV-1 infection".

Abstract needs to be re-written for clarity. It starts with virion host shut-off protein of herpes simplex virus 1 degradation effect on translated mRNAs, then drifts away to circRNAs in herpes simplex virus infection, then again away from virion host shut-off protein effects to the other immediate-early herpes simplex virus proteins. At this point, it already is confusing: is it circRNA production being elevated, or linear production

suppressed, or there is degradation of linear RNA? It then goes on to influenza virus, which has completely different mechanisms, to show similar effects. Further, there is no clear segregation of results, hypotheses, assumptions and conclusions in the abstract. Perspectives and placement of the results on the global scope are also absent.

We have rewritten the abstract according to the reviewer's suggestions.

In Introduction, line 43 "CircRNAs form closed RNA-loops" – very confusing as overlaps with another broadly accepted term "RNA closed-loop" which is a loop and has nothing to do with a perfect covalent circle of circRNAs.

We revised this sentence to start with "CircRNAs form covalent RNA circles…". Furthermore, new Fig 1a now outlines briefly biogenesis of circRNAs.

Further, some studies that support the role of ICP22 and ICP27 on the splicing patterns of NEAT1_2 and/or NEAT1_1 could be mentioned. Nice mention of specific roles of all the proteins involved in the pathway.

The corresponding studies, which support a potential role of ICP22 or ICP27 on splicing of NEAT1_2, are already mentioned in the introduction, but we now also reference them briefly in the discussion. However, as we also note in the discussion, induction of NEAT1_2 splicing by co-expression of ICP22 and ICP27 could simply be due to up-regulation of NEAT1_2 and correspondingly reduced competition between paraspeckle proteins and splicing factors for NEAT1_2 binding.

Please also note that, except for siRNA-mediated depletion of CDK7 and MED1, splicing of NEAT1_2 is restricted to sequences unique to NEAT1_2. Thus, NEAT1_1 does not appear to be spliced. We clarified this in the manuscript.

Furthermore, the authors have touched the roles of splicing of NEAT1_1 and NEAT1_2 splicing in HSV1, are the roles similar in influenza A? What does the previous literature state about this?

There is no previous literature on the roles of NEAT1_2 splicing in HSV-1 or influenza A infection since this is the first study to report on this. We can thus only hypothesize on the role of NEAT1_2 splicing in either HSV-1 or influenza A infection. NEAT1 is up-regulated in both HSV-1 and influenza A infection and NEAT1_2 plays a general antiviral role by mediating IL-8 up-regulation. This argues for similar mechanisms or roles of NEAT1_2 splicing in HSV-1 and influenza A infection. However, NEAT1 also plays a proviral role in HSV-1 infection by regulating HSV-1 gene expression and siRNA-mediated NEAT1 knockdown heals skin lesions caused by HSV-1 infection in mice. To our knowledge, no proviral role of NEAT1 in influenza A infection has been reported. Thus, the impact of NEAT1_2 splicing in HSV-1 and influenza A infection likely differs at least partly. We revised the discussion to extend on this aspect.

Not very clear in Methods how mapping and filtering was done. CircRNA detection relies on crossing the back-spliced junction in the approaches used. Thus, it is critical to understand how long were the insert sizes, how were thee treated and what happened to the reads with the 5 mismatches. How reliable was the remaining fully mapped overlap?

We revised the methods section of circRNA detection to provide more details on the algorithms and parameters used for the underlying alignment algorithms. Notably, the 5 mismatch threshold was only used for the linear RNA-seq mapping obtained with ContextMap2, which was used to remove candidate circular junction reads if they could be aligned linearly to the genome with up to 5 mismatches.

For circRNA detection, the number of allowed mismatches in the overlap on either side of the circular junction depends on the filtering approach used by the circRNA detection method, the underlying alignment algorithm, and the length of overlaps. For instance, CIRI2 combines pairs of clipped BWA alignments, each of which have to have an alignment score ≥ 19. With the default match score of 1 and a mismatch penalty of 4 used in BWA, this means that no mismatches are allowed for overlaps up to 23 bp, 1 mismatch is allowed for overlaps between 24 to 28 bp, 2 mismatches for overlaps of 29 to 33 bp, and so on.

We have provided more details on the approaches employed by the used algorithms to clarify this point, however, a full description of all algorithms is beyond the scope of this article and we refer the reader to the original manuscripts.

While supplementary figures are very numerous, I am not sure about the presentation approach and its logic. Are they necessary all? One does not need to include in the manuscript all what was plotted, just the bits that reinforce the conclusions made or hypotheses proposed.

Except for old S23 Fig, all Supplementary Figures contain additional information to which we refer to in the main manuscript, but generally only in a summarized form. We thus think that these Supplementary Figures are necessary to allow readers to look at the details of corresponding analyses and verify our statements in the manuscript if they want to. If they do not consider it necessary, they can ignore the corresponding Supplementary Figures. We thus kept all Supplementary Figures except for S1 Fig, which is now shown in revised Figs 1b and 2a, and S23 Fig, which was completely removed.

Discussion is one of the better-written parts of the manuscript, although some parts are more of a fit to Results-type and placement in the global picture and leads to subsequent investigations could have been unfolded more extensively. Results section needs re-writing for the clarity and flow.

We have revised the results section in order to clarify the issues raised by the reviewer.

Among some others, word “strikingly” is used far too often.

All uses of “strikingly” have been removed from the manuscript.

Almost all figures use very small fonts and have font size disparity in the panels – very often making the information illegible.

We have revised figures to increase fonts and harmonize font sizes between panels.

Figure 1 in the introduction sections can be moved into the discussion or results sections as this figure contains relatively detailed results/cases, instead of illustrating

the motivations behind the work. The authors may consider to include depiction of known role of cRNA in HSV-1, or potent role of NEAT1 and CDK7 in HSV-1, or even biogenesis of cRNA in relation to the specific circRNA in question.

Old Figure 1 has been split into two figures, with new Fig 1c containing the depiction of NEAT1 transcripts described in the introduction and new Fig 2 containing results. Thus, new Figure 2 was moved into the results section. New Fig 1 now contains additional panels outlining circRNA biogenesis (new Fig 1a) and experimental set-up of the HSV-1 infection time-courses (new Fig 1b, previously S1 Fig a).

We now also discuss the potential biogenesis of the NEAT1_2 circRNA in reference to general circRNA biogenesis outlined in Fig. 1a in the results section where the link between linear and circular splicing of NEAT1_2 is first reported.

A compact extract of material such as in Supplementary figure 1, 5 and 14 would be good to add in figure 1.

The outline of our *de novo* circRNA detection pipeline was moved from the Supplement (old S1 Fig 1b) into the main figures (new Fig 2a).

In Supplementary figure 18, it is best to use bars and not line plot – the measurement was not continuous.

This figure was changed to use barplots.

Reviewer #2:

The manuscript by Friedl et al investigates the effect of HSV1 infection on circular RNA formation using data previously generated by the group and uncovers an interesting effect of HSV1 on the linear and back splicing of the long NEAT1 isoform. The paper is clearly written, the study is methodologically thorough and the results are novel. I have a few comments and suggestions for improvement.

1. The circRNA detection pipeline uses a threshold of 2 reads/jusction for detecting circRNAs. Since sequencing depth varies considerably between samples (SFig2) – from 10 million to 100 million reads, this would strongly impact the number of circRNAs detected in each sample and likely explains the inter-sample variability. There also seems to be a bias in sequencing depth with the WT having less total reads than the ∆vhs in both total RNA and 4sU pulldown. The data in Fig1b (and related SuppFigs) should present numbers based on a threshold normalised to sequencing depth.

Fig 2b now shows numbers of identified circRNAs in total RNA with thresholds for circRNA identification adjusted to sequencing depth, i.e. the total number of human reads. Thus, for the sample with lowest sequencing depth a threshold of 2 reads is still used, while higher thresholds are used for samples with higher sequencing depth. The corresponding figure without adjusting thresholds is now S1 Fig a.

The following points are now also discussed in the corresponding results section: Normalized to sequencing depth, a smaller number of circRNAs was detected in mock infection for the Δ*vhs* infection time-course compared to the WT infection time-course. In part, this could be due to shorter read length (76 nt in the Δ*vhs* infection time-course vs. 101 nt in the WT infection time-course), which is also investigated in the manuscript. However, there also appear to be general differences in the number of circRNAs detected in different experiments, which can also be seen in the study referred to in comment #2 of this reviewer. To account for experimental differences in the number of circRNAs detected in the two time-courses, we thus always compared infected samples against mock infection of the same time-course to determine circRNA enrichments. Moreover, since the linear regression estimate of circRNA enrichment fits well to both highly and lowly expressed circRNAs, exclusion of lowly expressed circRNAs by adapting circRNA detection thresholds to sequencing depth would not alter our conclusions.

2. Line 144, the manuscript states "Consistent with previous reports [42] and the high stability of circRNAs, only a small number of circRNAs were detected in 4sU-RNA (119 and 194 in at least one sample of WT and Δvhs 4sU-seq, respectively)." I don't think the results are consistent with ref42 which found ~ 1800 circRNAs at 4h of 4sU abeling. We also find a substantial number of circRNAs in 4sU labelled samples at 1-2h. So the low number observed here is either due to low sequencing depth or perhaps cell-type specific?

While Zhang *et al.* (old ref42) indeed found ~1800 circRNAs after 4 h of 4sU labeling, the numbers of circRNAs recovered after 1 h labeling (the labeling duration used in our two time-courses) are smaller with 255, 820, 876 circRNAs in PA1, hESC H9 and H9 differentiated FB Neurons cells, respectively. They also conclude that "efficiency of circRNA processing from pre-mRNA is very low in cells", which is consistent with our

results. We have extended this part in the manuscript to provide more details. In particular, we discuss that the number of circRNAs identified by Zhang *et al.* in total RNA also varies considerably between cell types (6740, 4523, and 11185, respectively). This highlights a high variability between experiments and/or cell types in the number circRNAs detected. Moreover, the higher number of circRNAs detected by Zhang *et al.* can also partly be explained by the use of only one circRNA detection algorithm (CIRCexplorer). This is less restrictive than requiring independent identification of circRNAs by two algorithms as done in our study, but has a higher false positive rate. Combination of at least two distinct circRNA detection algorithms for *de novo* circRNA detection has previously suggested to improve reliability of circRNA detection (Hansen, Front. Cell Dev. Bio., 2018). Moreover, circRNA_finder and CIRI2 require presence of canonical GT-AG splicing signals, while CIRCexplorer also identifies potential circRNAs with other less common splicing signals.

3. The columns of Fig2a need labelling. Also please add a diagram of start and end coordinates for all the NEAT1 circRNAs.

Columns in heatmaps of circRNAs (now Fig 3a and Fig 5a) are now labeled with circBase IDs of corresponding circRNAs. Start and end coordinates for all identified NEAT1_2 circRNAs together with their circBase IDs are now shown in Fig 1c.

4. To convincingly prove the circularization of the NEAT1_2 isoform the authors could provide Sanger sequencing data following PCR with inverted primers flanking the BSJ.

We believe that we have provided sufficient evidence for circularization of the NEAT1_2 isoform as:
(1) Enrichment of the circRNA region compared to surrounding regions is observed both after treatment with RNase R (an exonuclease used to enrich circRNAs) and in HSV-1 infection. In the latter case, enrichment is due the high stability of circRNAs (due to resistance to exonuclease degradation) compared to the highly instable NEAT1_2 linear transcripts.
(2) Confirming read pairs can be recovered in significant numbers for this circRNA during HSV-1 infection. These are read pairs, where each individual read can be aligned linearly to the genome but the direction and relative orientation of the reads to each other is crosswise and not consistent with linear transcripts (outlined in S14 Fig c, results shown in S14 Fig b). This provides the same information as inverted primers, as these read pairs can only originate from circular RNAs. Furthermore, it allows easily validating the circRNA in the samples used for our analysis.
(3) Even when requiring an overlap of 40 bp on both sides of the junction, high numbers of circular junction reads can still be recovered in total and 4sU-RNA of WT HSV-1 infection. This excludes that circRNA identification is based only on short, potentially spurious overlaps.
(4) The sequences on both sides of the circular junction are unique in the host genome, excluding false positives due to repetitive sequences.

Minor
In Fig1b, SFig2a it would help to have the biological replicates next to each other.

These figures (new Fig 2b and S1 Fig a,b) have been adjusted to show replicates next to each other.

In Fig2b the track labelling is unclear. Two tracks have the same label “uninfected total” is one WT and one ∆vhs?

Track labeling in this figure (new Fig 3b) has been adjusted to clarify that these tracks show uninfected samples from the WT and ∆vhs infection time-course, respectively.

Reviewer #3:

This bioinformatic analysis reveals the presence of linear splicing and back splicing of NEAT1, leading to circular RNA formation for this abundant nuclear transcript normally found within paraspeckles. In the study the authors have used publicly available RNA-seq datasets from different cell types, in either un-infected, infected with wildtype HSV or influenza viruses, or various mutant viral strains that lack key components. They have interrogated these datasets for circular RNA species, using many prediction tools and normalising in different ways. The main findings are a discovery of a circular RNA derived from the long isoform of NEAT1; the finding that this isoform is also extensively linearly spliced; and the finding that these species increase in abundance when cells are infected with either HSV, or influenza viruses.

I come at this review, not as a bioinformatics expert, but as a scholar with expertise in NEAT1. Given this expertise I can attest that the manuscript is thoughtful and has incorporated the existing NEAT1 literature to a thorough degree. In particular, I appreciate the discussion including paraspeckle protein RBP CLIP datasets into the circular and spliced RNA models; consideration for existing observations on transcriptional inhibitors and RNA stability of NEAT1 and paraspeckles; and understanding of the semi-extractability of NEAT1. In terms of scientific accuracy for the findings – these appear to be convincing (with respect to my limited bioinformatic capability), as (1) many datasets are used, from different cell types and with different viruses, all showing the same result of circular NEAT1 and (2) multiple prediction tools are used to isolate the circRNA from the datasets. Of course, it would be nice to have orthogonal methods confirm the results (wet lab experimentation etc) however, these are not essential.

The only thing I would like to see changed is inclusion of a new dedicated paragraph in the discussion where the authors speculate about the effects of circRNA and linear NEAT1 splicing on the actual appearance of paraspeckles within cells. This is something that needs to be considered as some of the datasets where these spliced species are found are linked to prior data suggesting paraspeckles increase (for example with viral infection) and other datasets linked to a decrease in paraspeckles/paraspeckle disappearance (eg. DRB and Actinomycin D). Yet in both instances the spliced/backspliced forms of NEAT1 are apparent. Hence it would be informative to arrive at a unified model of how the spliced NEAT1 relates to paraspeckles as seen down the microscope.

We added sections to the discussion speculating about the effects of circRNA and linear NEAT1 splicing on the appearance of paraspeckles within cells. It should be noted though that in contrast to DRB treatment, Actinomycin D treatment does not lead to NEAT1 circular or linear splicing. This is now emphasized in the revised manuscript. In brief, we hypothesize that spliced NEAT1_2 transcripts cannot serve as a scaffold for paraspeckle assembly as binding sites for core paraspeckle proteins are spliced out and spatial organization of NEAT1_2 in paraspeckles is highly ordered. This could suggest that NEAT1_2 splicing contributes to paraspeckle disassembly upon DRB treatment and that paraspeckle disassembly upon DRB and Act-D treatment at least partly results from different mechanisms. Considering the up-regulation of NEAT1_2 upon both HSV-1 and influenza A infection, NEAT1_2 splicing may not abolish increased production of paraspeckles but only serve to dampen it. Alternatively,

however, splicing may only occur in HSV-1 and influenza A infection because more NEAT1_2 is available than paraspeckle proteins, making NEAT1_2 accessible to splicing factors. Nevertheless, we speculate that diffuseness of paraspeckles observed previously in influenza A infection (Imamura *et al.*, Molecular Cell, 2014) could at least be partly due to altered paraspeckle formation on spliced NEAT1_2 isoforms, as NEAT1_2 was not as strongly up-regulated upon influenza A infection as upon HSV-1 infection.
